# Enhancer of Polycomb and the Tip60 complex repress hematological tumor initiation by negatively regulating JAK/STAT pathway activity

**DOI:** 10.1242/dmm.038679

**Published:** 2019-05-30

**Authors:** Alessandro A. Bailetti, Lenny J. Negrón-Piñeiro, Vishal Dhruva, Sneh Harsh, Sean Lu, Aisha Bosula, Erika A. Bach

**Affiliations:** 1 Department of Biochemistry and Molecular Pharmacology, New York University School of Medicine, New York, NY 10016, USA; 2 Helen L. and Martin S. Kimmel Center for Stem Cell Biology, New York University School of Medicine, New York, NY 10016, USA

**Keywords:** JAK/STAT, Myeloproliferative neoplasms, *Drosophila*, E(Pc), Tip60, Melanotic tumors, Lysine acetyltransferases, Tumor suppressor

## Abstract

Myeloproliferative neoplasms (MPNs) are clonal hematopoietic disorders that cause excessive production of myeloid cells. Most MPN patients have a point mutation in *JAK2* (*JAK2^V617F^*), which encodes a dominant-active kinase that constitutively triggers JAK/STAT signaling. In *Drosophila*, this pathway is simplified, with a single JAK, Hopscotch (Hop), and a single STAT transcription factor, Stat92E. The *hop^Tumorous-lethal^* [*hop^Tum^*] allele encodes a dominant-active kinase that induces sustained Stat92E activation. Like MPN patients, *hop^Tum^* mutants have significantly more myeloid cells, which form invasive tumors. Through an unbiased genetic screen, we found that heterozygosity for *Enhancer of Polycomb* [*E(Pc)*], a component of the Tip60 lysine acetyltransferase complex (also known as KAT5 in humans), significantly increased tumor burden in *hop^Tum^* animals. Hematopoietic depletion of *E(Pc)* or other Tip60 components in an otherwise wild-type background also induced blood cell tumors. The *E(Pc)* tumor phenotype was dependent on JAK/STAT activity, as concomitant depletion of *hop* or *Stat92E* inhibited tumor formation. Stat92E target genes were significantly upregulated in *E(Pc)-*mutant myeloid cells, indicating that loss of *E(Pc)* activates JAK/STAT signaling. Neither the *hop* nor *Stat92E* gene was upregulated upon hematopoietic *E(Pc)* depletion, suggesting that the regulation of the JAK/STAT pathway by E(Pc) is dependent on substrates other than histones. Indeed, *E(Pc)* depletion significantly increased expression of Hop protein in myeloid cells. This study indicates that E(Pc) works as a tumor suppressor by attenuating Hop protein expression and ultimately JAK/STAT signaling. Since loss-of-function mutations in the human homologs of E(Pc) and Tip60 are frequently observed in cancer, our work could lead to new treatments for MPN patients.

This article has an associated First Person interview with the first author of the paper.

## Abstract (precis)

**Editor's choice:** Using *Drosophila* as a low-complexity model for human myeloproliferative neoplasms, the authors identified a conserved mechanism by which the Tip60 lysine acetyltransferase acts as a tumor suppressor by repressing JAK protein expression in a histone-independent manner.

## INTRODUCTION

Myeloproliferative neoplasms (MPNs) make up a group of clonal disorders of the myeloid lineage, including polycythemia vera (PV), essential thrombocythemia (ET) and primary myelofibrosis (PMF). A gain-of-function mutation in the Janus tyrosine kinase *JAK2* (*JAK2^V617F^*) is the most prevalent mutation in MPNs and accounts for ∼95% of PV cases and ∼60% of ET and PMF cases (Jones et al., 2005; Kralovics et al., 2005; Levine et al., 2005; James et al., 2005; Tefferi, 2016). The constitutively active JAK2^V617F^ kinase is ligand independent, and in animal models, its oncogenic potential depends on its downstream target STAT5, a member of the STAT transcription factor family (Walz et al., 2012; Yan et al., 2012; Sachs et al., 2016). Treatments for MPN patients, including phlebotomy, aspirin and JAK2 inhibitors, are temporary and not curative (Tefferi, 2016), highlighting the need for new treatments.

**Fig. 1.**
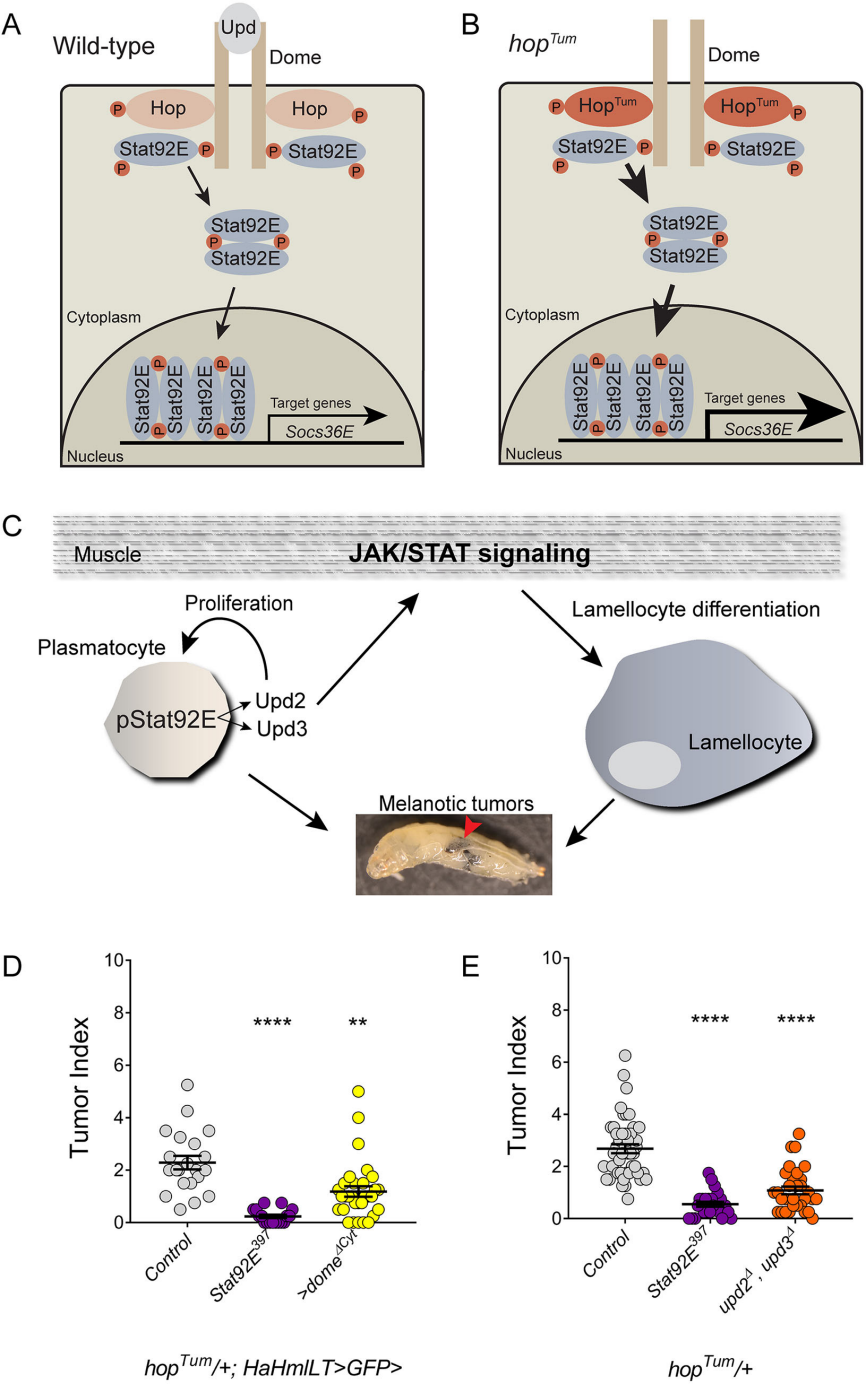
**Hop^Tum^ is a dominant-active kinase that causes hematopoietic tumors.** (A,B) Model of the *Drosophila* JAK/STAT pathway in wild-type (A) or *hop^Tum^* (B) blood cells. In wild-type (A), an Upd cytokine binds to a dimeric cell-surface receptor, Dome. This induces the transactivation of associated Hop tyrosine kinases. These activated Hop proteins then phosphorylate the Dome cytoplasmic domain. Inactive Stat92E proteins bind to the activated receptor, after which Stat92E becomes a substrate for Hop. Phosphorylated Stat92E dimers translocate to the nucleus, where they bind specific DNA sequences and alter gene transcription. A well-established Stat92E target gene is *Socs36E*. (B) Hop^Tum^ is a dominant-active kinase that activates Stat92E in a ligand-independent manner. This mutation leads to sustained activation of Stat92E, sustained transcriptional responses by activated Stat92E and increased expression of Stat92E target genes (bigger arrows). Brown circles with ‘P’ indicate tyrosine phosphorylation events. (C) Model of dysregulated hematopoiesis and melanotic tumor formation in *hop^Tum^* larvae. Stat92E activation (pStat92E) in plasmatocytes leads to the induction of Upd2 and Upd3. These cytokines are released into the hemolymph and activate JAK/STAT signaling in larval muscle. This in turn is required for the differentiation of lamellocytes, a key cell type in the formation of melanotic tumors. Upd2 and Upd3 can act in an autocrine manner to increase proliferation of plasmatocytes. The combination of ectopic differentiation of lamellocytes and the expansion of the plasmatocyte population leads to the formation of melanotic tumors in larval stages (red arrowhead). (D) Graph of the tumor index of *hop^Tum^* adult females of the indicated genotypes. The tumor index for *hop^Tum^* outcrossed to wild–type (*hop^Tum^*/+, *n*=21 for D and *n*=46 for E) are the gray circles in D,E. This tumor index is significantly suppressed by heterozygosity for *Stat92E* (*n*=17 for D and *n*=25 for E) using the null allele *Stat92E^397^* (D,E, purple circles). The tumor index of *hop^Tum^* adult females is also significantly suppressed when a dominant-negative version of Dome (Dome^ΔCyt^, *n*=31) is mis-expressed in the hematopoietic compartment (D, yellow circles) or when *hop^Tum^* females are also systemically heterozygous for both *upd2* and *upd3* deletions (*n*=31) (E, orange circles). The genotypes for the animals in panels D and E and all subsequent figures are listed in the supplemental information. Error bars represent s.e.m. **P<0.01; ****P<0.0001.

JAK2 and STAT5 are components of the conserved JAK/STAT signaling pathway, which regulates multiple developmental and immunological processes, including hematopoiesis (Levy, 1999; Amoyel et al., 2014). The pathway is triggered when extracellular ligands bind to cell-surface receptors, which activate receptor-associated JAKs. These kinases subsequently activate cytoplasmic STAT dimers through phosphorylation of a highly conserved C-terminal tyrosine residue (O'Shea et al., 2002). Phosphorylated STAT dimers translocate to the nucleus, bind to specific sites in genomic regulatory regions and alter target gene expression. In *Drosophila*, the JAK/STAT pathway is conserved but simplified, with three IL-6-like cytokines [Unpaired 1 (Upd1), Upd2 and Upd3], one Gp130-like cytokine receptor [Domeless (Dome)], one JAK [Hopscotch (Hop)] and one STAT (Stat92E) (Fig. 1A and Herrera and Bach, 2019). Activation of the *Drosophila* JAK/STAT pathway induces expression of target genes, such as *Socs36E*, *chinmo*, *zfh1*, *upd2* and *upd3* (Bach et al., 2007; Flaherty et al., 2010; Leatherman and Dinardo, 2008; Bazzi et al., 2018; Yang et al., 2015). The simplicity of the *Drosophila* JAK/STAT pathway represents an ideal model system to study JAK/STAT signaling *in vivo*.

Similar to vertebrates, *Drosophila* hematopoiesis occurs in two temporally distinct waves, the first during embryogenesis and the second during larval stages (reviewed in Gold and Brückner, 2015; Honti et al., 2014; Letourneau et al., 2016; Banerjee et al., 2019). In the embryo, multipotent hematopoietic progenitors called prohemocytes differentiate primarily into plasmatocytes, which function as macrophages in immunity, wound healing and tissue remodeling (Tepass et al., 1994; Wood and Jacinto, 2007). During larval stages, the embryonic plasmatocytes migrate to ‘hematopoietic pockets’, microenvironments located in each segment of the larval body wall (Markus et al., 2009; Makhijani et al., 2011). In pockets, the peripheral nervous system supports resident (or sessile) embryonic plasmatocytes, which self-renew and proliferate (Leitao and Sucena, 2015; Petraki et al., 2015). As a result, the embryonically derived pool of plasmatocytes increases 30-fold during larval stages. Sessile plasmatocytes are gradually released into circulation beginning in the second larval instar (Makhijani et al., 2011). However, they can be mobilized *en masse* in response to infection (Markus et al., 2009; Makhijani et al., 2011; Gold and Brückner, 2015). Additionally, in response to immune challenge, for example parasitization by ovidepository wasps, plasmatocytes can transdifferentiate into lamellocytes, large flat cells that encapsulate objects too large to be phagocytosed (Markus et al., 2009; Honti et al., 2010; Stofanko et al., 2010; Avet-Rochex et al., 2010; Anderl et al., 2016).

The second wave of hematopoiesis occurs in the larval lymph gland, an organ which serves as a reservoir of prohemocytes, which differentiate primarily into plasmatocytes during second and third larval instars (Tepass et al., 1994; Lebestky et al., 2000; Mandal et al., 2004; Jung et al., 2005). However, under immune-challenged conditions, lymph gland prohemocytes can also differentiate into lamellocytes (Jung et al., 2005; Rizki, 1978). The lymph gland disintegrates in early pupal stages, releasing mature hemocytes into circulation (Grigorian et al., 2011). Lineage-tracing studies have shown that the adult hemocyte pool is derived from both embryonic and lymph gland hematopoiesis (Holz et al., 2003).

The *hop^Tum^* mutation is a temperature-sensitive, gain-of-function mutation in the *Drosophila* JAK caused by a G341E substitution, which results in sustained activation of Stat92E (Fig. 1B and Luo et al., 1995; Harrison et al., 1995). This mutation causes a ‘fly leukemia’ characterized by the presence of melanotic tumors, black masses comprised of aggregated plasmatocytes and lamellocytes. Importantly, lamellocytes are always observed in genetic backgrounds harboring melanotic tumors (Zettervall et al., 2004; Minakhina and Steward, 2006). The *hop^Tum^* mutation is X-linked and is lethal in hemizygous males, and the melanotic tumors are manifest in heterozygous females (Corwin and Hanratty, 1976; Hanratty and Ryerse, 1981). Melanotic tumors appear black as a result of activation of the prophenol oxidase pathway, and the dysregulation of hematopoiesis in *hop^Tum^* larvae shares features with the larval immune response triggered by wasp-egg infestation (Minakhina and Steward, 2006; Yang and Hultmark, 2016).

Similar to MPN patients, the *hop^Tum^* leukemic phenotype originates in hematopoietic multipotent progenitors and can be adoptively transferred for multiple generations and up to 2 years (Hanratty and Ryerse, 1981). Tumors are not observed in this genotype until the middle of the third larval instar (Hanratty and Ryerse, 1981; Lanot et al., 2001). In *hop^Tum^* larvae, the stereotypic pattern of hematopoietic pockets is disrupted and sessile plasmatocytes are mobilized (Anderson et al., 2017). In *hop^Tum^* larvae, the lymph gland also prematurely histolyzes, releasing lymph-gland-derived plasmatocytes and lamellocytes into circulation (Hanratty and Ryerse, 1981; Lanot et al., 2001; Sorrentino et al., 2007; Anderson et al., 2017; Terriente-Félix et al., 2017). In *hop^Tum^* animals, the number of plasmatocytes and lamellocytes in circulation is dramatically increased, as result of upregulated plasmatocyte proliferation and massive induction of lamellocyte differentiation (Luo et al., 1995; Lanot et al., 2001; Silvers and Hanratty, 1984; Anderson et al., 2017; Bazzi et al., 2018). Sustained activation of the JAK/STAT pathway in plasmatocytes induces *upd2* and *upd3*, which encode pathway agonists (Fig. 1C). These ligands then activate the JAK/STAT pathway non-autonomously in body wall muscle (Yang et al., 2015; Bazzi et al., 2018; Yang and Hultmark, 2017). JAK/STAT activation in muscle is required for the full maturation and function of lamellocytes in response to wasp infection, but the mechanism by which this occurs is not yet known (Yang et al., 2015).

By means of an F1 deficiency (Df) screen, we recently identified 32 enhancer Dfs and 11 suppressor Dfs that modified the tumor burden of *hop^Tum^* animals (Anderson et al., 2017). *Df(2R)ED2219* was among the strongest enhancers (Anderson et al., 2017). Here, we demonstrate that the gene uncovered by this Df and responsible for the enhancement of the *hop^Tum^* leukemic phenotype is *Enhancer of Polycomb* [*E*(*Pc*)]. Despite its name, E(Pc) is not a component of Polycomb group complexes but rather is part of the Tip60 complex, a member of the MYST family of lysine acetyltransferases (KATs). Although E(Pc) is not a catalytic subunit of Tip60, it is a critical co-factor, and physical interactions between E(Pc) and Tip60 regulate acetyltransferase activity (Searle et al., 2017; Xu et al., 2016). As KATs modify lysine residues in histones and other proteins, these enzymes regulate numerous processes, including protein stability/turnover, chromatin remodeling and tumorigenesis (Sheikh and Akhtar, 2019). The Tip60 complex can act as a tumor suppressor in human cancers. Mono-allelic loss of the human *TIP60* gene (*KAT5*) is a frequent event in mammary and head-and-neck carcinomas and in human lymphoma (Gorrini et al., 2007; Zack et al., 2013). Tip60 expression is also significantly downregulated in colon and lung carcinoma (Lleonart et al., 2006). Furthermore, other loss-of-function mutations (point mutations, deletions and other genetic aberrations) in human *KAT5*, or in human *EPC1* or *EPC2* [the human homologs of *E(Pc)*] are observed in numerous studies of myeloid and lymphoid malignancy in cBioPortal.org (Cerami et al., 2012; Gao et al., 2013). However, the causal factors that are dysregulated in human cancer upon downregulation of the TIP60 complex are largely unknown.

Here, we show that *E(Pc)* heterozygosity significantly enhances the tumor burden in *hop^Tum^* animals, similar to the effect of the enhancing deficiency *Df(2R)ED2219* that uncovers it. Furthermore, we find that *E(Pc)* and *Tip60* are required in the hematopoietic compartment to repress myeloid lineage dysregulation and inhibit melanotic tumor formation. The oncogenic potential of *E(Pc)* and *Tip60* depends on the presence of *hop* and *Stat92E*, as concomitant depletion of either gene with *E(Pc)* or *Tip60* severely perturbs tumor formation. Furthermore, loss of *E(Pc)* or *Tip60* leads to cell-autonomous increases in the activity of the Stat92E protein and the expression of Stat92E target genes but does not alter expression of the *hop* or *Stat92E* genes. Finally, depletion of *E(Pc)* significantly increases the levels of Hop protein expression. Our model indicates that E(Pc) and the Tip60 complex [E(Pc)/Tip60] act as tumor suppressors by attenuating JAK/STAT signaling through repressing expression of the Hop protein.

## RESULTS

### Loss of E(Pc) or Tip60 enhances *hop^Tum^* tumors

As mentioned above, plasmatocytes that harbor the Hop^Tum^ protein upregulate expression of the pathway ligands *upd2* and *upd3*. These cytokines activate the JAK/STAT pathway non-autonomously in body wall muscle, and we reasoned that they may also have autocrine functions in plasmatocytes. To test this hypothesis, we blocked pathway activation in the hematopoietic compartment by mis-expressing a dominant-negative Dome receptor. We used the pan-hematopoietic driver *HaHmlLT-Gal4*, which is expressed strongly in circulating and sessile hemocytes, the larval lymph gland and pericardial cells (Mondal et al., 2014; Anderson et al., 2017). We also observed occasional expression in small patches of cells in the salivary gland and fat body (not shown), but it is not expressed in larval muscle (Anderson et al., 2017). Indeed, when we inhibit pathway activation in plasmatocytes, we significantly impair the formation of melanotic tumors in *hop^Tum^* animals (Fig. 1D, yellow circles). These results support a model in which the production of Upd cytokines by plasmatocytes in *hop^Tum^* larvae drives the formation of melanotic tumors by autonomous and non-autonomous mechanisms (Fig. 1C). Consistent with these observations, reducing the systemic level of *Stat92E* or both *upd2* and *upd3* significantly suppresses melanotic tumors in *hop^Tum^* animals (Fig. 1E, orange circles; Luo et al., 1997; Hou et al., 1996; Yan et al., 1996; Bazzi et al., 2018).

**Fig. 2.**
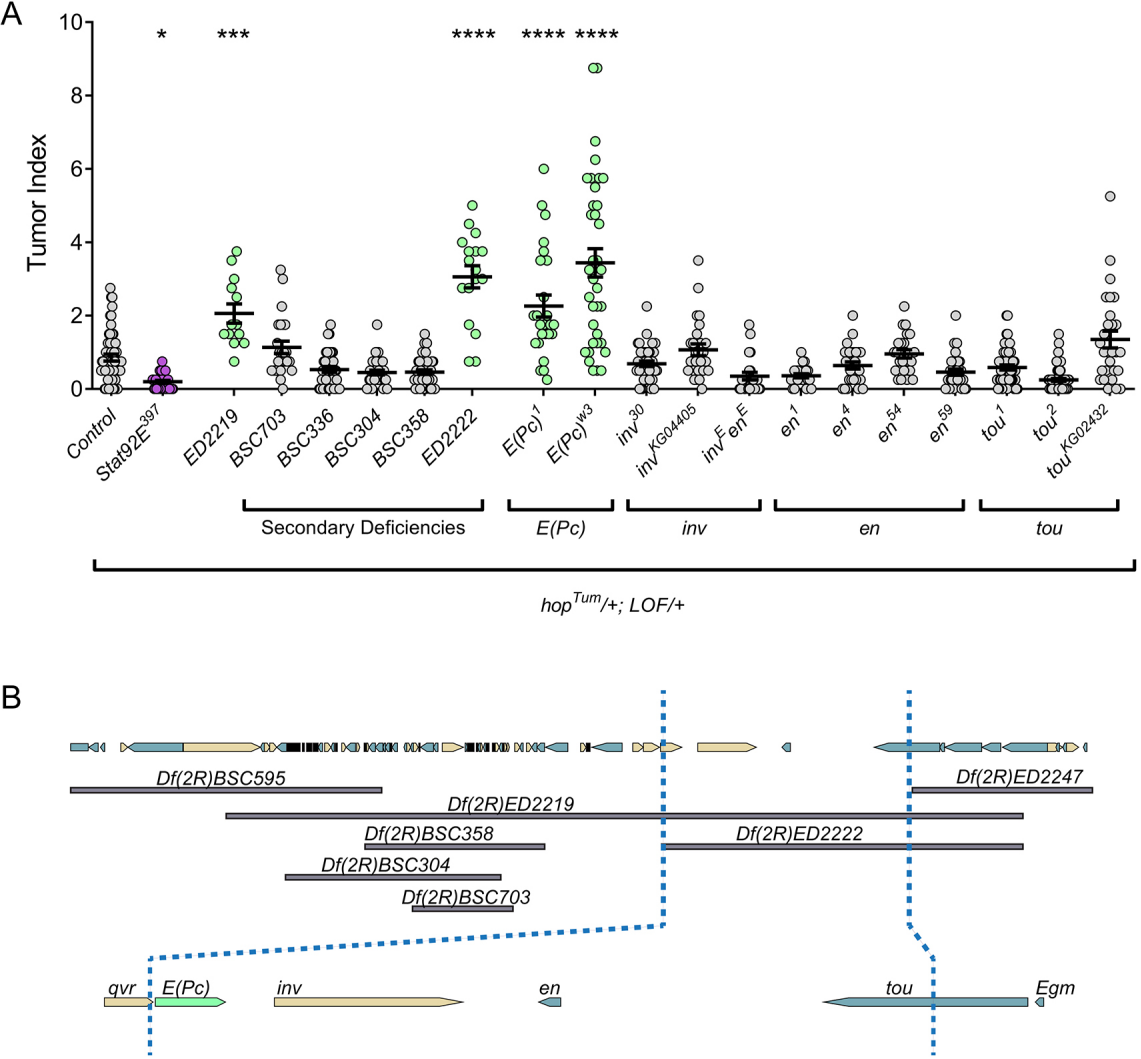
**Heterozygosity for *E(Pc*), or the overlapping deficiency *Df(2R)ED2219*, enhances *hop^Tum^* tumorigenicity*.*** (A) Graph of the tumor index of the indicated genotypes. The tumor index for *hop^Tum^* outcrossed to wild-type (*n*=55) is the first set of gray circles. This value is significantly suppressed by concomitant heterozygosity for *Stat92E* mutation (*n*=34; purple circles). *Df(2R)ED2219* heterozygosity (*n*=13) significantly enhances the tumor index (first set of green circles). Of the five Dfs that overlap with *Df(2R)ED2219* [*Df(2R)BSC703* (*n*=24), *Df(2R)BSC336* (*n*=36), *Df(2R)BSC304* (*n*=33), *Df(2R)BSC358* (*n*=41), *Df(2R)ED2222* (*n*=17)], only *Df(2R)ED2222* enhances the *hop^Tum^* tumor index (second set of green circles). Of the four genes uncovered by *Df(2R)ED2219* and *Df(2R)ED2222* [*E(Pc)*, *inv*, *en*, *tou*], only alleles of *E(Pc)* enhanced the tumor index (third and fourth set of green circles). *E(Pc)^1^* (*n=*25); *E(Pc)^w3^* (*n*=36); *inv^30^* (*n*=41); *inv^KG04405^* (*n*=26); *inv^E^en^E^* (*n*=23); *en^1^* (*n*=23); *en^4^* (*n*=27); *en^54^* (*n*=23); *en^59^* (*n*=29); *tou^1^* (*n*=50); *tou^2^* (*n*=73); *tou^KG02432^* (*n*=28). Error bars represent s.e.m. *P<0.05; ***P<0.001; ****P<0.0001. (B) Model of the deletions and genes scored in panel A. The *E(Pc)* gene is shown in light green.

We previously performed a deficiency screen to identify dominant modifiers of *hop^Tum^* tumors (Anderson et al., 2017). One of the most robust enhancers in this screen was *Df(2R)ED2219* (Fig. 2A). To determine the underlying gene responsible for this modification, we surveyed five deficiencies that overlapped *Df(2R)ED2219* (Fig. 2B). Of these deficiencies, only *Df(2R)ED2222* significantly enhanced the *hop^Tum^* tumor phenotype (Fig. 2A). The genomic region common to *Df(2R)ED2219* and *Df(2R)ED2222* uncovered four genes: *E(Pc)*, *invected* (*inv*), *engrailed* (*en*) and *toutatis* (*tou*) (Fig. 2B). Using loss-of-function alleles for each candidate gene, we determined that only *E(Pc)* hypomorphic alleles significantly enhanced the *hop^Tum^* tumor phenotype (Fig. 2A). These data strongly implicate *E(Pc)* as being the gene responsible for the enhancement caused by *Df(2R)ED2219* heterozygosity. Owing to male lethality, we did not screen Dfs on the X chromosome for interactions with *hop^Tum^*, and as a result, we were not able to determine whether the Dfs uncovering the X-linked gene *Tip60* interacted in the screen (Anderson et al., 2017). Instead, we used RNA interference (RNAi) and mis-expression of a dominant-negative Tip60 (see below).

**Fig. 3.**
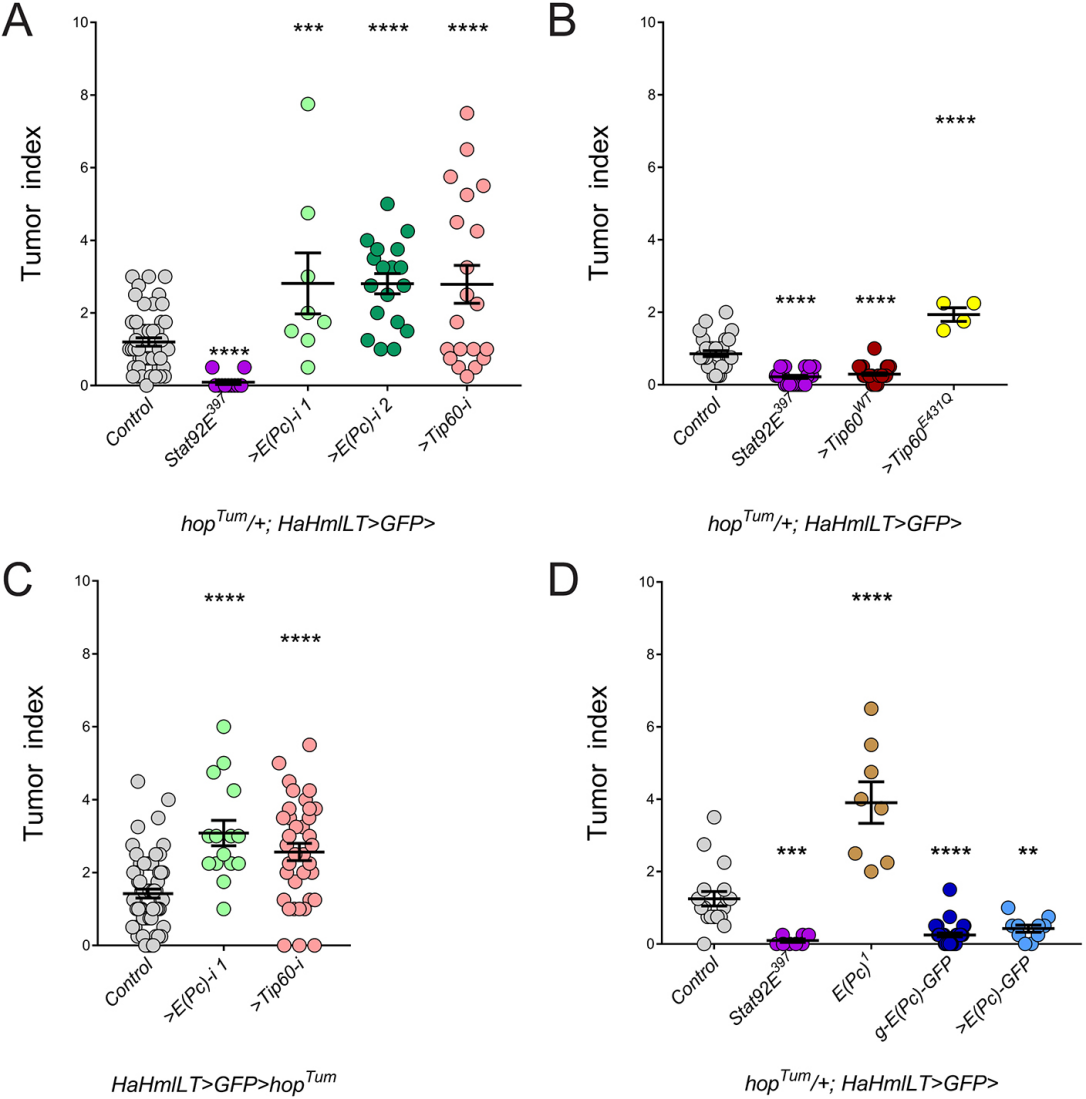
**E(Pc) works through the Tip60 complex to repress *hop^Tum^* melanotic tumors.** (A) In the endogenous *hop^Tum^* background (gray circles, *n*=50), hematopoeitic depletion of *E(Pc)* using the *GD12282* line [light green circles, labeled *>E(Pc)-i 1*, *n*=8] or using the *JF03101* line [dark green circles, labeled *>E(Pc)-i 2*, *n*=18)] or *Tip60* (pink circles, *n*=20) enhances the tumor phenotype. The purple circles represent the suppression of tumor burden by heterozygosity for *Stat92E* mutation (*n*=11). (B) Increasing the dose of *Tip60* by mis-expressing a wild-type version (*>Tip60^WT^*, *n*=34; dark red circles) significantly reduces the tumor burden in *hop^Tum^* (endogenous allele, *n*=30; gray circles). By contrast, mis-expressing a dominant-negative *Tip60* (*>Tip60^E431Q^*, *n*=4; yellow circles) significantly enhances the tumor burden in *hop^Tum^* (endogenous allele) animals. We note that very few adult female *hop^Tum^/+; HaHmlLT>Tip60^E431Q^* eclose, suggesting pupal lethality. The purple circles represent the suppression of tumor burden by heterozygosity for *Stat92E* mutation (*n*=24). (C) Mis-expression of *UAS-hop^Tum^* (*n*=64) in the hematopoietic compartment using the *HaHmlLT-Gal4* driver induces melanotic tumors (gray circles). Concomitant depletion of *E(Pc)* using the *GD12282* line [green circles, labeled *>E(Pc)-i 1*, *n*=15] or *Tip60* (pink circles, *n*=36) significantly increases the tumor burden in these animals. (D) Increasing the dose of *E(Pc)* by supplying an additional genomic copy [*g-E(Pc)-GFP*, *n*=33] or by mis-expressing a wild-type *E(Pc)* tagged with GFP [*>E(Pc)-GFP*, *n*=10] (dark and light blue circles, respectively) significantly reduces the tumor burden in endogenous *hop^Tum^* animals (gray circles, *n*=18), similar to heterozygosity for *Stat92E* mutation (purple circles, *n*=8). By contrast, heterozygosity for the *E(Pc)^1^* loss-of-function allele (brown circles, *n*=8) significantly enhances the tumor burden in endogenous *hop^Tum^* animals. Error bars represent s.e.m. **P<0.01; ***P<0.001; ****P<0.0001.

We next assessed whether the *E(Pc)* global heterozygous phenotype was actually due to loss of *E(Pc)* in the hematopoietic compartment. We significantly depleted *E(Pc)* or *Tip60* using the hematopoietic driver *HaHmlLT-Gal4* (Fig. S1). Hematopoietic depletion of either *E(Pc)* or *Tip60* significantly increased the *hop^Tum^* tumor phenotype (Fig. 3A, green and pink circles), as did mis-expression of dominant-negative *Tip60^E431Q^* (Fig. 3B, yellow circles). We previously established that mis-expression of *UAS-hop^Tum^* by *HaHmlLT-Gal4* induced lamellocytes in a cell-autonomous manner (Anderson et al., 2017). It is important to note that mis-expression of wild-type Stat92E alone does not induce JAK/STAT target genes (Ekas et al., 2010). To further confirm that the *E(Pc)*/*Tip60* phenotype is autonomous to the hematopoietic lineage, we concomitantly depleted either gene while mis-expressing *UAS-hop^Tum^.* Either manipulation resulted in a significant enhancement of the tumor burden (Fig. 3C, green and pink circles). We next addressed the converse: whether increasing the dose of wild-type *E(Pc)* or wild-type *Tip60* would suppress the *hop^Tum^* tumor phenotype. We increased the *E(Pc)* genomic dose using a GFP-tagged genomic copy [*g-E(Pc)-GFP*] or mis-expressed a wild-type version of either factor. In all three scenarios, increasing the level of wild-type *E(Pc)* or *Tip60* significantly suppressed the *hop^Tum^* tumor phenotype and to the same degree as halving the genetic dose of *Stat92E* (Fig. 3B,D). *hop^Tum^*/*Y* males normally die before adulthood (Fig. S2, gray bar), but they eclose in considerable numbers when they are systemically heterozygous for *Stat92E*, or when *Stat92E* or *hop* are hematopoietically depleted (Fig. S2, purple, green and yellow bars, respectively). Remarkably, increasing hematopoietic expression of *E(Pc)* or *Tip60* also rescued *hop^Tum^*/*Y* adult males (Fig. S2, blue and red bars). Since these surviving *hop^Tum^* males, regardless of manipulation, still contain melanotic tumors, it is not clear mechanistically how hematopoietic depletion of *hop* or *Stat92E*, or hematopoietic mis-expression of *E(Pc)* or *Tip60*, rescues them. Taken together, these results indicate that *E(Pc)* and *Tip60* act in the hematopoietic lineage to repress *hop^Tum^* tumor formation and *hop^Tum^*/*Y* male lethality.

### Hematopoietic depletion of *E(Pc)* or *Tip60* in wild–type animals leads to ectopic lamellocyte differentiation and melanotic tumors

**Fig. 4.**
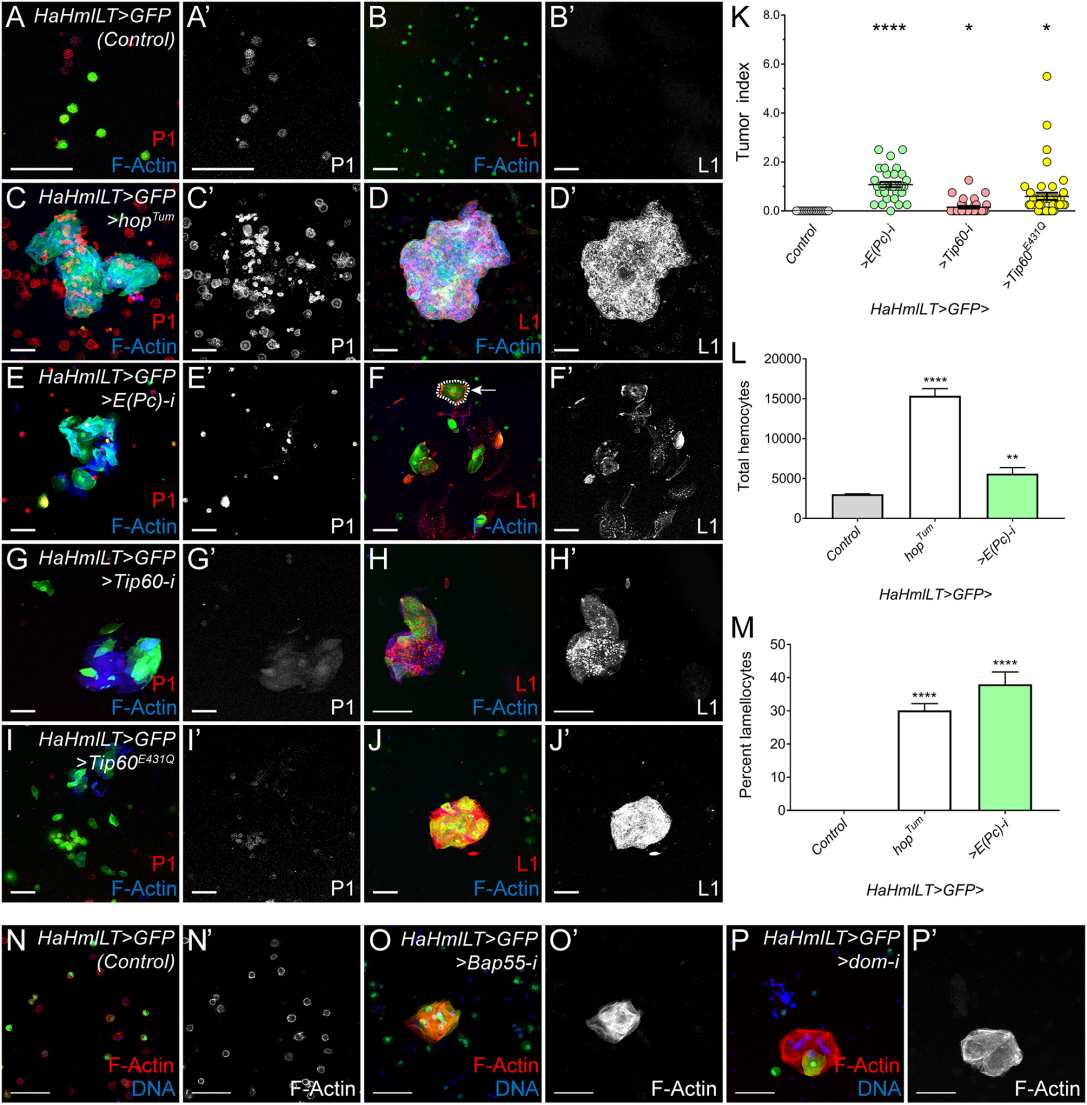
**Hematopoietic depletion of *E(Pc)/Tip60* induces precocious lamellocyte differentiation and tumor formation.** (A-B′) Bleeds from control larvae (*HaHmlLT>GFP*) contain P1-positive plasmatocytes (red, A) but not L1-positive lamellocytes (red, B). (C-D′) Larvae with hematopoietic mis-expression of *UAS-hop^Tum^* have an increase in plasmatocytes (C) and an ectopic differentiation of lamellocytes (D). (E-J) Hematopoietic depletion of *E(Pc)* (E,F) or *Tip60* (G,H) or hematopoietic mis-expression of a dominant-negative *Tip60^E431Q^* (I,J) in an otherwise wild-type background induces lamellocyte differentiation. F-actin is blue in A-J. Experiments in A-J were performed at least three times with similar results. (K) Graph of tumor indices from adult females. Melanotic tumors are not observed in control (*HaHmlLT>GFP*) animals (gray circles, labeled ‘Control’, *n*=20). *E(Pc)* or *Tip60* depletion using the same driver increases the tumor burden (green and pink circles, respectively, *n*=33 for both genotypes). Mis-expression of *Tip60^E431Q^* also leads to melanotic tumors in wild-type animals (yellow circles, *n*=47). Error bars represent s.e.m. *P<0.05; **P<0.01; ****P<0.0001. (L,M) Hemocyte counts in controls, after hematopoietic mis-expression of *UAS-hop^Tum^* or hematopoietic depletion of *E(Pc)*. Hemocytes were counted from at least 15 individual larvae per genotype. (L) The average estimated total number of circulating hemocytes per larva. Hematopoietic mis-expression of *hop^Tum^* or hematopoietic depletion of *E(Pc)* led to a significant increase in the average total number of hemocytes per larva compared to the control *HaHmlLT>GFP*. (M) The corresponding percentage of lamellocytes. There were no lamellocytes in control larvae, but there were substantially more lamellocytes upon hematopoietic mis-expression of *hop^Tum^* or hematopoietic depletion of *E(Pc)*. (N-P) Hematopoietic depletion of the *Tip60* complex components *Bap55* (O) or *dom* (P) induces lamellocyte differentiation as assessed by F-actin (red) and the formation of microtumors. The control is in N. DNA is blue. Scale bars: 50 μm.

In wild-type, healthy larvae, the pool of circulating hemocytes comprises primarily plasmatocytes; lamellocytes are not observed (Honti et al., 2014). However, after immune challenge, lamellocytes quickly differentiate from multipotent progenitors or transdifferentiate from plasmatocytes (Honti et al., 2010; Jung et al., 2005; Anderl et al., 2016). To assess the role of *E(Pc)* and *Tip60* in hemocyte development, we depleted either factor in an otherwise wild-type background using the *HaHmlLT-Gal4* driver. In hemolymph bleeds from control larvae that only expressed the driver, we observed plasmatocytes but not lamellocytes (Fig. 4A,B). By contrast, in bleeds from animals hematopoietically mis-expressing *hop^Tum^*, we found an expansion of plasmatocytes and ectopic differentiation of lamellocytes (Fig. 4C,D; see the outlined cell, arrow, in 4F for an example of a lamellocyte). Furthermore, in this background, we observed the formation of clusters of lamellocytes and plasmatocytes (Fig. 4C,D), hereafter referred to as ‘microtumors’ (see Fig. 4D for an example and Materials and Methods for a description of microtumors). Strikingly, when we depleted *E(Pc)* or *Tip60* or mis-expressed dominant-negative Tip60^E431Q^ in wild-type hemocytes, we detected plasmatocytes, lamellocytes and microtumors in larval bleeds and frank melanotic tumors in adults (Fig. 4E-K). To determine whether hematopoietic depletion of *E(Pc)* increases the number of larval hemocytes or the number of lamellocytes, or both, we counted circulating hemocytes from control larvae (*HaHmlLT>GFP/+*), larvae with hematopoietic mis-expression of *hop^Tum^* (*HaHmlLT>hop^Tum^*), or larvae with hematopoietic depletion of *E(Pc)* (*HaHmlLT>E(Pc)RNAi*). Control larvae had ∼3000 estimated total circulating hemocytes and no lamellocytes, as expected (Fig. 4L,M). By contrast, *HaHmlLT>hop^Tum^* larvae had significantly more estimated total circulating hemocytes (∼15,000) and 30% lamellocytes (Fig. 4L,M), consistent with a prior report (Zettervall et al., 2004). In *HaHmlLT>E(Pc)RNAi*, the estimated number of total hemocytes was significantly increased (∼5600) compared to controls, and the percentage of lamellocytes was dramatically increased to 38% (Fig. 4L,M). These results indicate that the *E(Pc)* RNAi tumors are caused primarily by ectopic lamellocyte differentiation and secondarily by increased plasmatocyte proliferation.

We also determined that depletion of seven other Tip60 components, including *Brahma associated protein 55kD* (*Bap55*) and SWI2/SNF2 family member *domino* (*dom*), from the hematopoietic compartment, caused ectopic differentiation of lamellocytes and the formation of microtumors (Fig. 4N-P and Table 1). These phenotypes are consistent with prior reports of ectopic lamellocyte differentiation upon *E(Pc)* knockdown in lymph gland prohemocytes and in *dom* mutant larvae (Owusu-Ansah and Banerjee, 2009; Braun et al., 1998). These data indicate that the E(Pc)/Tip60 complex represses lamellocyte differentiation and tumor formation in wild-type animals.

**Table 1.**
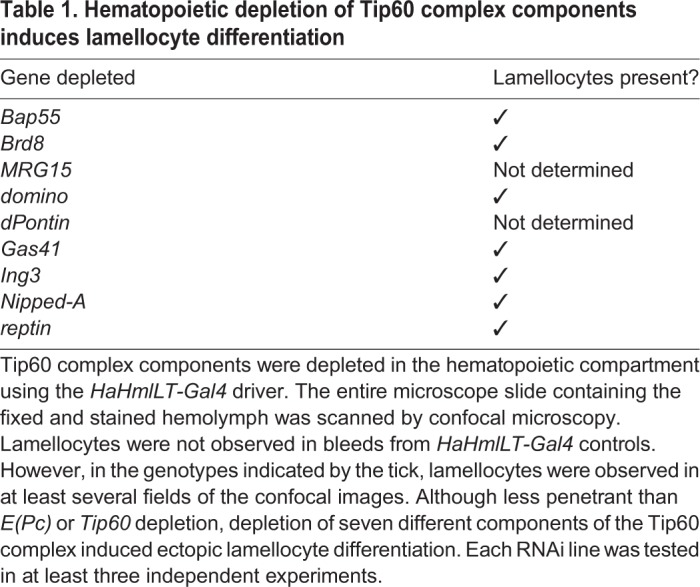
**Hematopoietic depletion of Tip60 complex components induces lamellocyte differentiation**

### *E(Pc)* acts upstream of or in parallel to *hop* in tumor formation

**Fig. 5.**
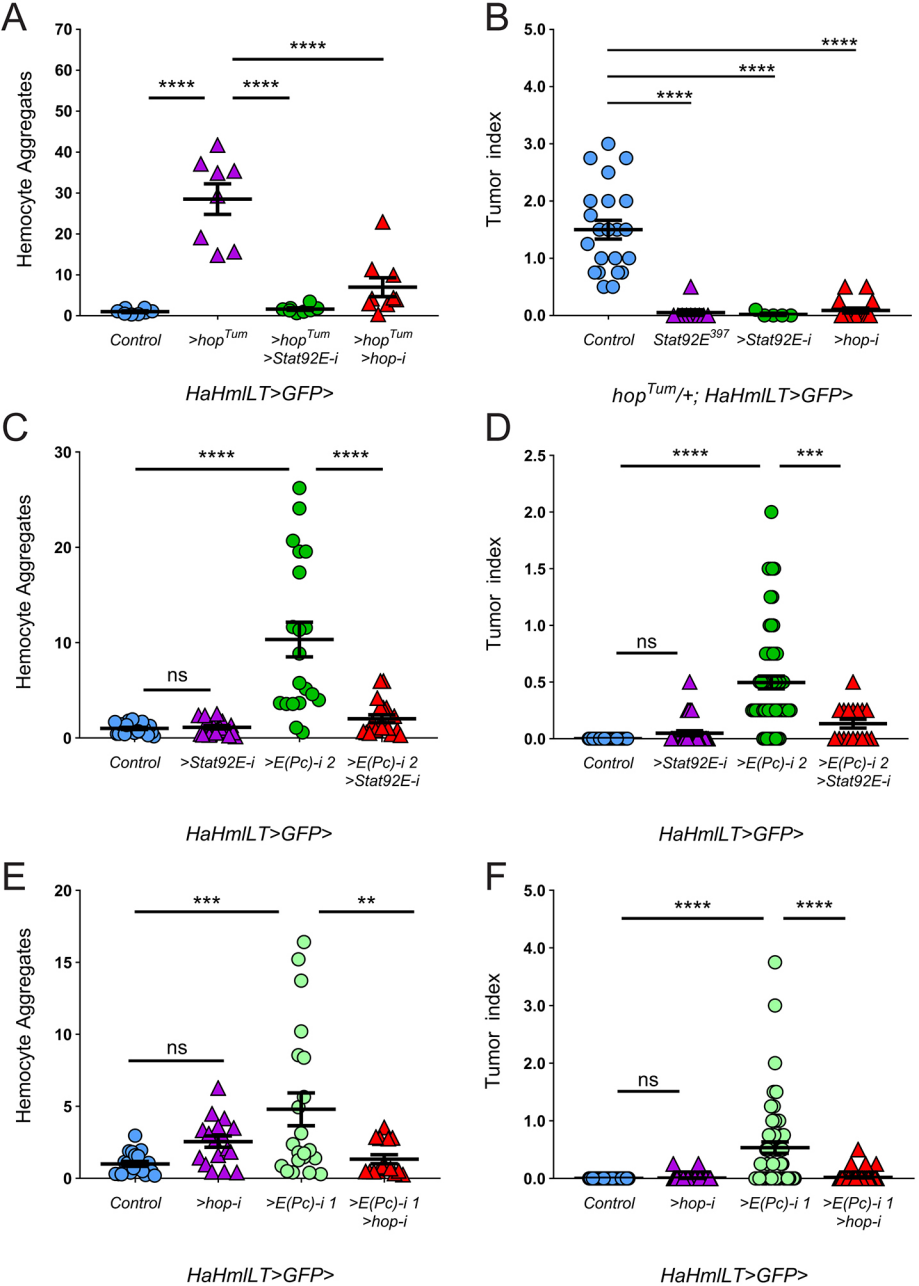
**The *E(Pc)*-depletion phenotype is abrogated by concomitant knockdown of *Stat92E* or *hop*.** (A,C,E) Graphs of larval hemocyte aggregation. (B,D,F) Graphs of adult tumor indices. (A) Hemocyte aggregation is significantly increased in *HaHmlLT>hop^Tum^* (purple triangles, *n*=8) compared to controls (blue circles, *n*=8). Depletion of *Stat92E* (green circles, *n*=8) or *hop* (red triangles, *n*=9) in *HaHmlLT>hop^Tum^* larvae significantly reduces hemocyte aggregation. (B) The adult tumor index of endogenous *hop^Tum^*/+ (blue circles, *n*=22) is significantly suppressed when *Stat92E^397^* is heterozygous (purple triangles, *n*=10), or when *Stat92E* or *hop* is hematopoietically depleted (green circles and red triangles, *n*=5 and 17, respectively). (C,D) Hematopoietic depletion of *E(Pc)* [green circles, labeled *E(Pc)-i 2*, *n*=20 for C and *n*=63 for D] in an otherwise wild-type background significantly increases hemocyte aggregation (C) or adult tumor burden (D) compared to control (blue circles, *n*=21 for C and *n*=40 for D). Concomitant depletion of *Stat92E* and *E(Pc)* (red triangles, *n*=19 for C and *n*=17 for D) significantly reduces hemocyte aggregation compared to depletion of only *E(Pc)*. There is no significant difference between the control (blue circles) and depletion of only *Stat92E* (purple triangles, *n*=19 for C and *n*=31 for D). (E,F) Hematopoietic depletion of *E(Pc)* [green circles, labeled *E(Pc)-i 1*, *n*=21 for E and *n*=57 for F] significantly increases hemocyte aggregation (E) or adult tumor burden (F) compared to control (blue circles, *n*=20 for E and *n*=55 for F). Concomitant depletion of *hop* suppresses hemocyte aggregation due to *E(Pc)* depletion (red triangles, *n*=15 for E and *n*=69 for F). There was no significant difference between the control (blue circles) and depletion of only *hop* (purple triangles, *n*=17 for E and *n*=90 for F). Error bars represent s.e.m. **P<0.01; ***P<0.001; ****P<0.0001; ns, not significant.

We developed an assay in which we quantified the GFP fluorescence intensity of aggregated hemocytes in the larval circulatory system as a proxy for tumor formation (see Materials and Methods). Bleeds from *HaHmlLT*>*hop^Tum^* larvae had significantly higher GFP intensity compared to control bleeds (Fig. 5A, purple triangles), and these aggregates consisted of GFP-labeled plasmatocytes and lamellocytes (Fig. 4D). Depletion of *Stat92E* or *hop* significantly decreased hemocyte aggregates in *HaHmlLT*>*hop^Tum^*, demonstrating that these aggregates result from increased JAK/STAT activity (Fig. 5A, green circles and red triangles, respectively). Furthermore, we showed that hematopoietic depletion of *Stat92E* or *hop* significantly reduced the tumor burden in animals heterozygous for the endogenous *hop^Tum^* allele (Fig. 5B, green circles and red triangles, respectively). These results document the efficacy of the *Stat92E* and *hop* RNAi transgenes in suppressing both larval hemocyte aggregates and adult melanotic tumors. As expected, hemocyte aggregation in control animals was minimal (Fig. 5C,E, blue circles), and depletion of *Stat92E* or *hop* in an otherwise wild-type background also produced minimal aggregation (Fig. 5C,E, respectively, purple triangles). Hematopoietic depletion of *E(Pc)* significantly increased hemocyte aggregation in larvae and tumor burden in adults (Fig. 5C-F, green circles). To test whether the *E(Pc)* RNAi hemocyte aggregation and tumor phenotypes depended on JAK/STAT signaling, we concomitantly depleted *E(Pc)* and *Stat92E* or *E(Pc)* and *hop*. Indeed, knockdown of either *Stat92E* or *hop* significantly suppressed both phenotypes (Fig. 5C-F, red triangles). These data indicate that tumor initiation caused by *E(Pc)* depletion is dependent on the JAK/STAT pathway.

### JAK/STAT signaling is repressed by the E(Pc)/Tip60 complex

**Fig. 6.**
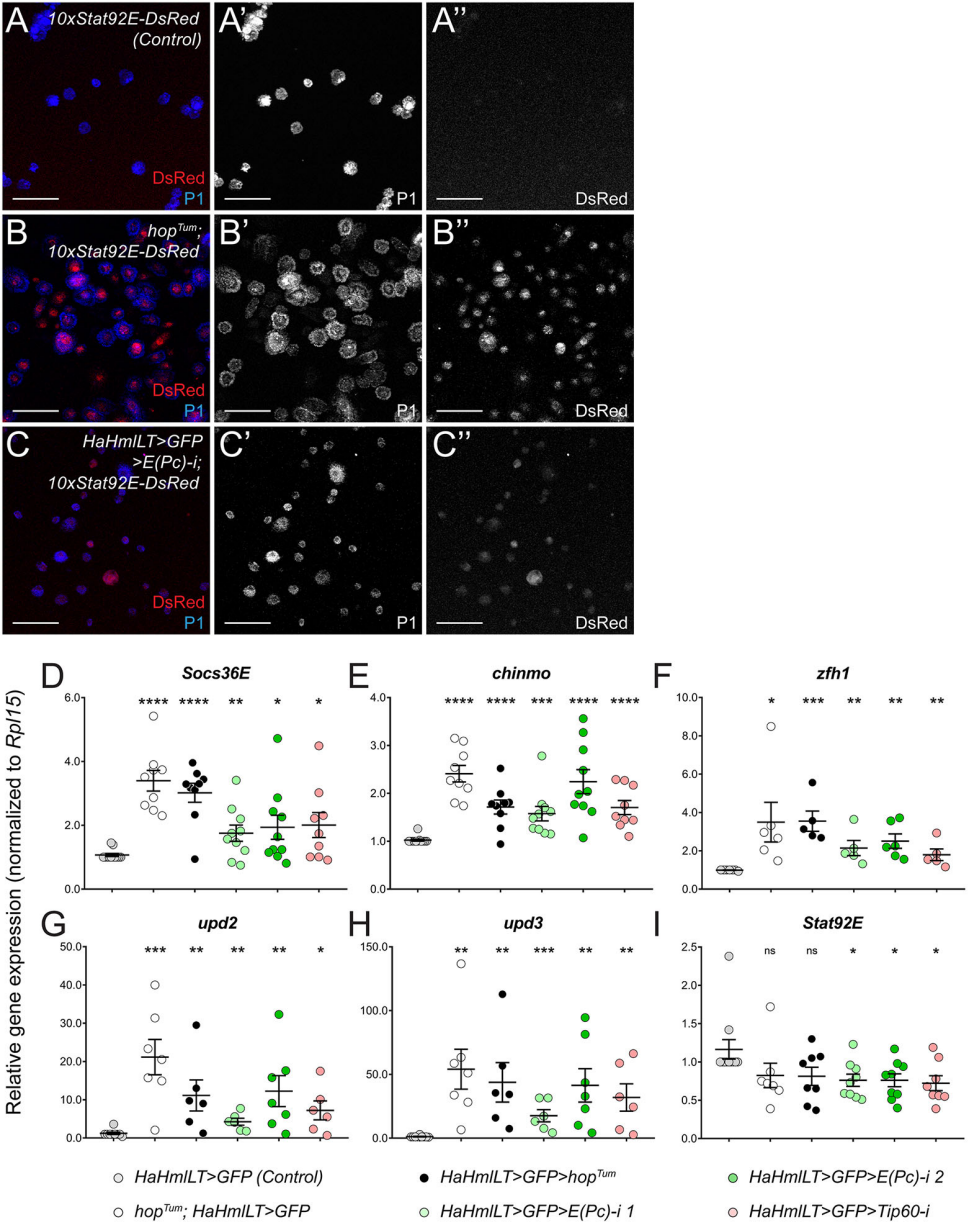
**E(Pc)/Tip60 autonomously represses Stat92E activity.** (A) P1-positive plasmatocytes (blue) from bleeds of control larvae display a low level of Stat92E activity as monitored by 10xSTAT-DsRed (red). (B) P1-positive plasmatocytes (green) from bleeds of *hop^Tum^* larvae display a high level of Stat92E activity as monitored by 10xSTAT-DsRed (red). (C) P1-positive plasmatocytes (green) from larvae hematopoietically depleted for *E(Pc)* display high Stat92E activity as monitored by 10xSTAT-DsRed (red). Scale bars: 50 μm. Experiments in A-C were performed at least three times with similar results. (D-I) qPCR on mRNAs from circulating hemocytes from these larvae: control (gray circles), endogenous *hop^Tum^/+* (white circles); *UAS-hop^Tum^* (black circles); *E(Pc) RNAi 1* (light green circles); *E(Pc) RNAi 2* (dark green circles); *Tip60 RNAi* (pink circles). (D-F) Transcription of Stat92E target genes, *Socs36E* (D), *chinmo* (E) and *zfh1* (F) are significantly increased in hemocytes from endogenous *hop^Tum^*, *UAS-hop^Tum^*, *E(Pc) RNAi* (using either RNAi construct) and *Tip60 RNAi* compared to the control. For D and E, *n*=12 (control); *n*=9 (*hop^Tum^/+*); *n*=9 (*UAS-hop^Tum^*); *n*=10 [*E(Pc)-i 1*]; *n*=10 [*E(Pc)-i 2*]; *n*=9 (*Tip60-i*). For F, *n*=7 (control); *n*=6 (*hop^Tum^/+*); *n*=9 (*UAS-hop^Tum^*); *n*=5 [*E(Pc)-i 1*]; *n*=6 [*E(Pc)-i 2*]; *n*=5 (*Tip60-i*). (G,H) Transcription of *upd2* (G) and *upd3* (H) are significantly upregulated in hemocytes in endogenous *hop^Tum^*, *UAS-hop^Tum^*, *E(Pc) RNAi* (using either RNAi construct) and *Tip60 RNAi* compared to the control. For G and H, *n*=9 (control); *n*=7 (*hop^Tum^/+*); *n*=6 (*UAS-hop^Tum^*); *n*=6 [*E(Pc)-i 1*]; *n*=7 [*E(Pc)-i 2*]; *n*=6 (*Tip60-i*). (I) Transcription of the *Stat92E* gene is not increased in any of the genotypes tested. Rather, *Stat92E* mRNA is significantly decreased in *UAS-hop^Tum^*, *E(Pc) RNAi* (using either RNAi construct) and *Tip60 RNAi*, but not endogenous *hop^Tum^*, compared to the control. For I, *n*=11 (control); *n*=7 (*hop^Tum^/+*); *n*=8 (*UAS-hop^Tum^*); *n*=9 [*E(Pc)-i 1*]; *n*=9 [*E(Pc)-i 2*]; *n*=8 (*Tip60-i*). Error bars represent s.e.m. *P<0.05; **P<0.01; ***P<0.001; ****P<0.0001; ns, not significant.

Our results thus far raised the possibility that the E(Pc)/Tip60 complex regulates activity of the JAK/STAT pathway. To address this issue, we assessed whether *E(Pc)* depletion could cell-autonomously increase Stat92E activation. We monitored Stat92E activity using an established Stat92E transcriptional reporter (*10xStat92E-DsRed*) containing regulatory sequences from a Stat92E target gene, *Socs36E*, to drive expression of the fluorescent protein DsRed (Bach et al., 2007). Control hemocytes from uninfected wild-type animals displayed low levels of DsRed (Fig. 6A)*.* As expected, this reporter was strongly upregulated in hemocytes from *hop^Tum^* larvae (Fig. 6B). Upon *E(Pc)* hematopoietic depletion, we also observed a robust induction of *10xStat92E-DsRed* (Fig. 6C). Furthermore, the Stat92E target genes *Socs36E*, *chinmo* and *zfh1* were significantly upregulated in hemocytes upon depletion of *E(Pc)* or *Tip60* (Fig. 6D-F, green and pink circles). The *upd2* and *upd3* genes were induced in hemocytes in the endogenous *hop^Tum^* background as well as upon hematopoietic mis-expression of *UAS*-*hop^Tum^* (Fig. 6G,H, white and black circles), consistent with a recent report (Bazzi et al., 2018). Both *upd2* and *upd3* were also significantly upregulated in hemocytes depleted for *E(Pc)* or *Tip60* (Fig. 6G,H, green and pink circles). Importantly, transcription of the *Stat92E* gene was not increased in *E(Pc)/Tip60*-depleted hemocytes, ruling out the model that E(Pc)/Tip60 negatively regulates JAK/STAT activity by repressing the *Stat92E* gene (Fig. 6I, green and pink circles). Taken together, these data demonstrate that, in wild-type hemocytes, E(Pc)/Tip60 negatively regulates JAK/STAT activity, thereby restricting lamellocyte differentiation.

### Hematopoietic depletion of E(Pc) increases Hop protein expression *in vivo*

**Fig. 7.**
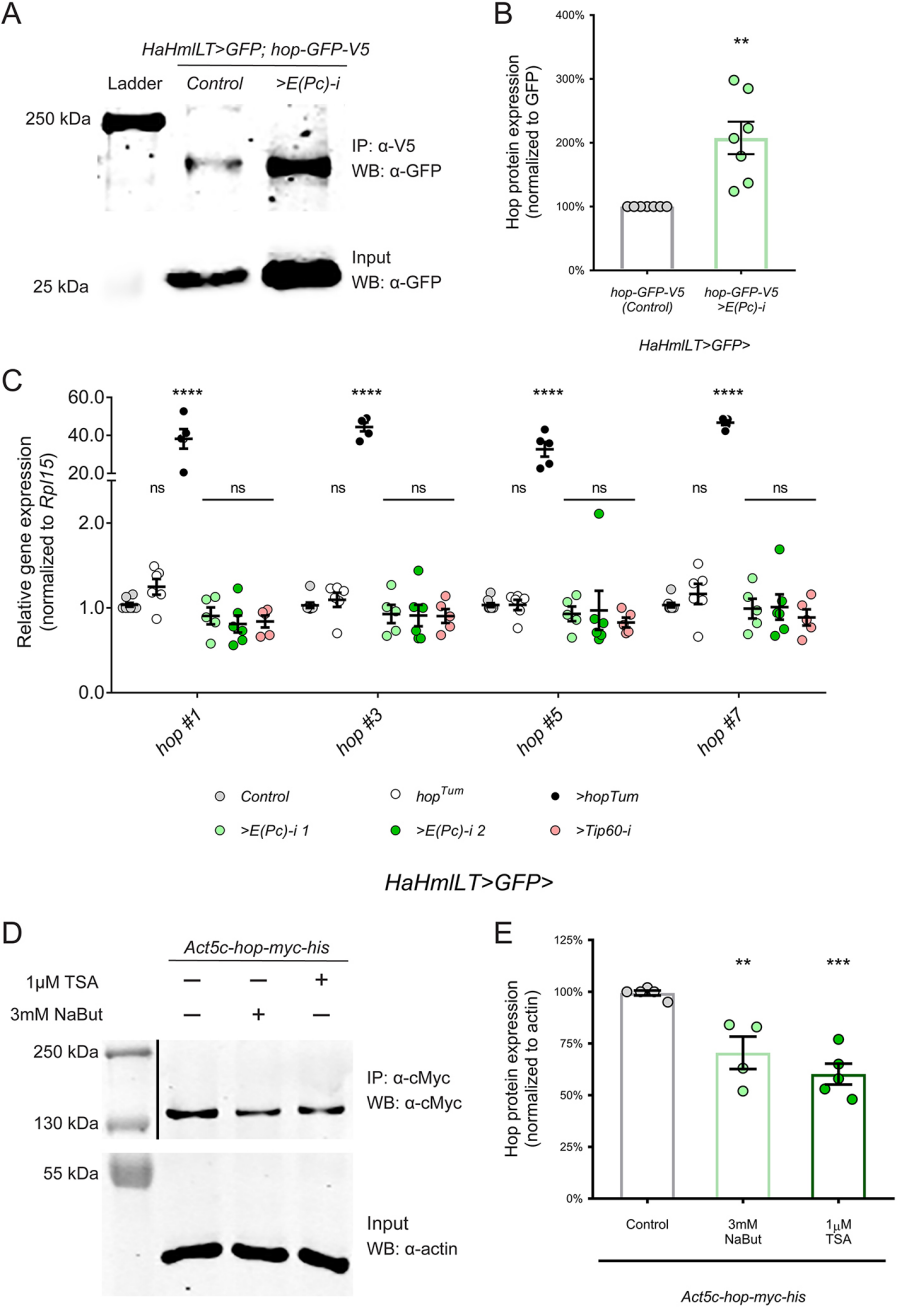
**E(Pc) depletion in the hematopoietic compartment increases Hop protein expression.** (A) Western blot with anti-GFP or anti-V5 immunoprecipitates for the Hop-GFP-V5 protein (top panel) or GFP from total cell lysates (bottom panel) from control and *E(Pc)*-depleted hemocytes. Hop-GFP-V5 is ∼150 kDa and GFP is 27 kDa. This experiment was performed seven times independently, with similar results. A representative western blot is shown here. (B) Graph of Hop-GFP-V5 protein normalized to the GFP input in the total cell lysate for control and *E(Pc)*-depleted hemocytes. Hop-GFP-V5 protein is significantly increased in *E(Pc)*-depleted hemocytes compared to control hemocytes. *N*=7 independent experiments. (C) qPCR on mRNAs from circulating hemocytes from these larvae: control (gray circles, *n*=8), endogenous *hop^Tum^/+* (white circles, *n*=6); *UAS-hop^Tum^* (black circles, *n*=5); *E(Pc) RNAi 1* (light green circles, *n*=6); *E(Pc) RNAi 2* (dark green circles, *n*=6); *Tip60 RNA-i* (pink circles, *n*=5). Expression of the *hop* gene is not increased in hemocytes from endogenous *hop^Tum^* or depleted *E(Pc)* and *Tip60*. As expected, when *hop^Tum^* is mis-expressed, *hop* mRNA is significantly increased. (D) S2 cells were transfected with *Act5c-hop-cMyc-His* and treated with 3 mM NaBut and 1 µM TSA for 16 h. Cells were lysed 48 h post-transfection. Western blotting of Hop-Myc-His immunoprecipitates (top blot) was performed using an anti-Myc antibody and the input (bottom blot) using an anti-actin antibody. This experiment was performed five times independently, with similar results. A representative western blot is shown here. (E) Graph of normalized Hop-Myc-His for each treatment. NaBut and TSA treatments significantly reduced Hop-Myc-His expression by 29.5% and 39.8%, respectively. *N*=5 independent experiments. Error bars represent s.e.m. **P<0.01; ***P<0.001; ****P<0.0001; ns, not significant.

Our genetic studies demonstrate that loss of *E(Pc)/Tip60* (i.e. decrease in lysine acetylation) leads to an increase in JAK/STAT signaling. Inhibitors of lysine deacetylases (termed KDACi) significantly inhibited proliferation of MPN cells and reduced disease burden in a preclinical mouse model of PV (Guerini et al., 2008; Akada et al., 2012). These data suggest that lysine acetyltransferases could regulate the level of Hop protein. We tested this hypothesis by measuring Hop protein expression using an epitope-tagged *hop-GFP-V5* expressed under endogenous regulatory sequences on a bacterial artificial chromosome in transgenic flies (Sarov et al., 2016). We isolated circulating hemocytes from control *hop-GFP-V5* larvae or from experimental *hop-GFP-V5* larvae in which *E(Pc)* was hematopoietically depleted. Hop-GFP-V5 protein was normalized to expression of the 27 kDa GFP protein driven by *HaHmlLT>GFP.* In seven independent experiments, we observed a significant, 2-fold increase in the level of Hop-GFP-V5 protein in *E(Pc)-*depleted hemocytes compared to controls (Fig. 7A, quantified in 7B). We assessed whether the increase in Hop protein expression was due to increased transcription of the *hop* gene. We performed qPCR analysis using four independent pairs of primers expanding exon junctions of the *hop* transcript. As expected, hemocytes from the endogenous *hop^Tum^* mutant did not have a significant increase in *hop* transcription (Fig. 7C, white circles). By contrast, in the positive control, hemocytes mis-expressing *UAS*-*hop^Tum^* had a significant upregulation of *hop* expression (Fig. 7C, black circles). Importantly, *E(Pc)-* or *Tip60-*depleted hemocytes did not display any alteration in *hop* gene expression (Fig. 7C, green and pink circles). These results strongly suggest that E(Pc) negatively regulates Hop protein levels in *Drosophila* blood cells. To determine whether the converse is true, we assessed whether KDACi treatment would reduce Hop protein levels. We transfected hemocyte-derived S2 cells with a Myc epitope-tagged Hop (Hop-Myc-His), and then treated the cells with two different KDACi [1 µM trichostatin A (TSA) or 3 mM sodium butyrate (NaBut)] for 16 h. In five independent experiments, we observed a significant reduction in the level of Hop–Myc protein in KDACi-treated S2 cells compared to vehicle DMSO-treated controls (Fig. 7D, quantified in 7E). Hop protein was reduced by 29.5% with 3 mM NaBut treatment and by 39.8% with 1 µM TSA treatment. Taken together, these data indicate that the activity of the lysine acetyltransferase E(Pc)/Tip60 directly or indirectly regulates the level of Hop protein in *Drosophila* blood cells, thereby controlling the level of JAK/STAT pathway activity.

## DISCUSSION

In this study, we characterized the deficiency *Df(2R)ED2219*, which significantly enhanced the tumor burden in *hop^Tum^* animals (Anderson et al., 2017). After testing additional deficiencies that overlapped with *Df(2R)ED2219*, we found that only *Df(2R)ED2222* recapitulated this enhancement. These two overlapping deficiencies uncovered four genes, only one of which – *E(Pc)* – enhanced the *hop^Tum^* tumor phenotype when heterozygous. We found that the enhancement caused by loss of *E(Pc)* was due to its role in the hematopoietic system through the Tip60 KAT complex. Furthermore, we showed that E(Pc) is critical for repressing the precocious differentiation of lamellocytes, that this repression is JAK/STAT dependent, and that the depletion of *E(Pc)* causes a substantial increase in JAK/STAT signaling by increasing levels of the Hop protein. This leads to increased hemocyte production of Upd2 and Upd3 by activated Stat92E, which then presumably triggers JAK/STAT signaling non-autonomously in muscle and increases lamellocyte differentiation by means of a currently unknown mechanism. As a result, melanotic tumors are significantly larger in *hop^Tum^* animals that are heterozygous for an *E(Pc)* mutation compared with *hop^Tum^* heterozygous for a wild-type allele.

A recent publication reported that E(Pc) repressed expression of the *Stat92E* gene by directly binding regions near the transcription start site in somatic cells of the *Drosophila* testis (Feng et al., 2017). While we cannot rule out the possibility that the reduction of E(Pc)/Tip60 causes precocious lamellocyte differentiation as a result of changes in histone acetylation, we did not observe a change in *Stat92E* transcripts upon depletion of *E(Pc)/Tip60*. Therefore, the *E(Pc)/Tip60* loss-of-function phenotype in the hematopoietic compartment is not a result of ectopic expression of the *Stat92E* gene. We do observe cell-autonomous increases in the expression of Stat92E target genes upon *E(Pc)/Tip60* depletion, arguing that increased Stat92E activity occurs upon loss of *E(Pc)/Tip60* in hemocytes. Nevertheless, future work will be needed to determine whether E(Pc)/Tip60 acts on chromatin and/or histones at Stat92E target genes or at genes that regulate pathway activity. Loss of E(Pc)/Tip60 appears to elicit cell-type-specific responses. For example, Tip60 interacts with the transcription factor Myc to maintain *Drosophila* neural stem cells (Rust et al., 2018), and regulates expression of CyclinB or germline differentiation genes in germline cells in the *Drosophila* ovary (McCarthy et al., 2018; Feng et al., 2018).

**Fig. 8.**
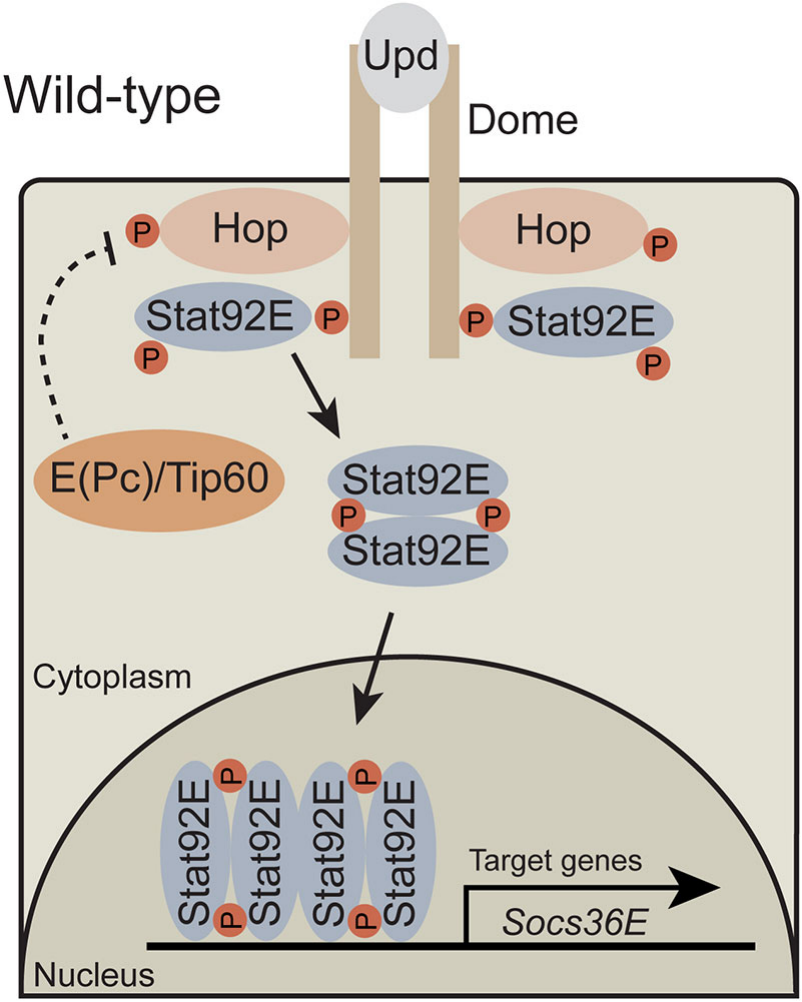
**Model of JAK/STAT being regulated by signaling by E(Pc)/Tip60.** E(Pc)/Tip60 suppresses lamellocyte differentiation and tumor formation. E(Pc)/Tip60 represses JAK/STAT signaling by inhibiting the protein expression of Hop through a direct or indirect (dotted lines) mechanism. See text for details.

Our unbiased genetic screen identified mutations in *E(Pc)* as potent enhancers of hematopoietic tumors in *Drosophila*, indicating that this phenotype is sensitive to the reciprocal activity of KATs and KDACs. We favor the interpretation that E(Pc)/Tip60 negatively regulates the levels of Hop protein either directly or indirectly and that this regulatory mechanism is a causal event in melanotic tumor formation in *hop^Tum^*. Our model (Fig. 8) is supported by several lines of evidence. First, numerous groups have reported that upregulation of Hop^WT^ or Hop^Tum^ protein is sufficient to induce melanotic tumors, indicating that Hop protein levels are causal to this oncogenic phenotype (Harrison et al., 1995; Zettervall et al., 2004; Luo et al., 1995; Bazzi et al., 2018; Anderson et al., 2017). Second, depletion of *hop* significantly reduces tumor formation caused by loss of *E(Pc)*. Third, the level of Hop protein in purified larval blood cells is significantly increased upon depletion of *E(Pc)*. Fourth, the level of Hop protein in cultured S2 cells is significantly decreased upon treatment with KDACi. Lastly, the E(Pc)/Tip60 complex does not act on the *hop* gene: hematopoietic depletion of complex components does not increase *hop* transcripts. Currently, we do not know whether the E(Pc)/Tip60 complex regulates Hop protein expression at the level of translation of the *hop* mRNA, or at the level of Hop protein stability and/or turnover. Future work will be needed to address these issues.

As noted above, KDACi, which increase lysine acetylation, were highly effective in inhibiting proliferation of MPN cells and in reducing disease burden in a preclinical PV mouse model (Guerini et al., 2008; Akada et al., 2012). The chromatin-independent effects of KDACi in MPN cells result in large part from deacetylation of the chaperone Hsp90, of which JAK2 is a client protein (Bali et al., 2005; Wang et al., 2009; LaFave and Levine, 2012). Treatment of MPN cell lines with pan-KDACi panobinostat disrupts the interaction between Hsp90 and JAK2^V617F^ (Wang et al., 2009). Additionally, the Hsp90 inhibitor PU-H71 is effective at degrading JAK2^V617F^ in MPN cell lines and primary patient samples (Marubayashi et al., 2010). Moreover, administration of PU-H71 in preclinical PV and ET mouse models normalized blood counts, reduced allele burden and increased mean survival (Marubayashi et al., 2010). It is intriguing to speculate that a similar regulatory network exists between Hsp83, the *Drosophila* ortholog of Hsp90, and Hop in *Drosophila* myeloid-like cells, but future experiments will be needed to test this hypothesis. In sum, our results reveal remarkable similarities between the regulation of JAK proteins by KATs and KDACs across species and highlight the power of *Drosophila* as a low-complexity model for human MPNs.

## MATERIALS AND METHODS

### Fly stocks and husbandry

The following stocks were obtained from the Bloomington *Drosophila* Stock Center: *Stat92E^397^*; *hop^Tum^*; *Df(2R)ED2219*; *Df(2R)BSC703*; *Df(2R)BSC336*; *Df(2R)BSC304*; *Df(2R)BSC358*; *Df(2R)ED2222*; *E(Pc)^1^*; *E(Pc)^w3^*; *inv^30^*; *inv^KG04405^*; *inv^E^ en^E^*; *en^1^*; *en^4^*; *en^54^*; *en^59^*; *tou^1^*; *tou^2^*; *tou^KG02432^*; *upd2^Δ^ upd3^Δ^*; *E(Pc)* TRiP RNAi line *JF03101* [termed *E(Pc)-i* *2*]; *Tip60* TRiP RNAi line *HM05049*; *hop* TRiP RNAi line *GL00305*; *Bap55* TRiP RNAi line *HMS04015*; and the *dom* TRiP RNAi lines *HMS00192*, *HMS01855* and *HMS02208.*

The following RNAi stocks were acquired from the Vienna *Drosophila* RNAi Center: *E(Pc)^GD12282^* (v35268) [termed *E(Pc)-i 1*]; *Stat92E^GD4492^* (v43866); *Bap55^GD11955^* (v24703); *Brd8^GD8354^* (v41530), *Brd8^KK107830^* (v110618); *dMRG15^GD11902^* (v43802); *dMRG15^KK107689^* (v107689); *dom^GD1420^* (v7787); *dPontin^KK101103^* (v105408); *Gas41^GD4100^* (v12616); *Gas41^KK101151^* (v106922); *Ing3^GD11989^* (v52510); *Ing3^KK107543^* (v109799); *Nipped-A^GD15595^* (v40789); *reptin^GD4651^* (v19021); and *reptin^KK105732^* (v103483).

The *UAS-Dome^ΔCyt^* stock was a gift of James Castelli-Gair Hombria, Centro Andaluz de Biología del Desarrollo (Brown et al., 2001). The *E(Pc)-genomic-GFP*, *UAS-E(Pc)* and *UAS-E(Pc)-GFP* stocks were gifts from Dr Xin Chen, Johns Hopkins University (Feng et al., 2017). The *UAS-Tip60^WT^* and *UAS-Tip60^E431Q^* stocks were gifts of Dr Felice Elefant, Drexel University (Lorbeck et al., 2011). The *Hand-Gal4, HmlΔ-Gal4, UAS-FLP.JD1, UAS-2xEGFP; Gal4-Act5C (FRT.CD2)* (referred to as *HaHmlLT-Gal4*) stock was a gift from Dr Utpal Banerjee, University of California, Los Angeles (Mondal et al., 2014). We also used *UAS-hop^Tum^* (Anderson et al., 2017). *10x-Stat92E-DsRed* was a gift of Dr Martin Zeidler, Sheffield University, UK.

### Tumor indices

Tumor indices were scored as described in Anderson et al. (2017). We crossed *hop^Tum^* females to males that carried deficiencies or alleles. For each experiment, we set up in parallel: (1) a cross of *hop^Tum^*/*FM7* virgins to *Ore^R^* males, the progeny of which were used as a baseline control, and (2) a cross of *hop^Tum^*/*FM7* virgins to *Stat92E^397^/TM6B, Tb* males, the progeny of which were used to mark suppression of the tumor phenotype. We scored the melanotic tumors in adult F1 progeny according to how many quarters of the adult abdominal segments they encompassed. For instance, if a tumor covered one quarter of a segment, it was given a score of 0.25, whereas a tumor that covered one entire abdominal segment was given a score of 1.0. Each individual progeny was given a tumor index (TI), which is the sum of all tumor sizes per animal. The TI of each genotype corresponds to the average of all individual TIs in that genotype. TIs were graphed with standard error bars by GraphPad Prism 7. The minimum sample size of each genotype was 15, and all crosses were repeated at least three times.

### Antibodies

For immunohistochemistry, we used mouse anti-P1 (for plasmatocytes at 1:10) or mouse anti-L1 (for lamellocytes at 1:10), both gifts from Dr Istvan Andó (Kurucz et al., 2007). We used ToPro (Thermo Fisher Scientific) to label DNA at 1:1000 and phalloidin (Thermo Fisher Scientific) to label F-actin at 1:25. Fluorescent secondary antibodies were obtained from Jackson ImmunoResearch and used at 1:200. For immunoprecipitation, we used 1 µl/sample of mouse anti-V5 monoclonal (Thermo Fisher Scientific, #R960-25) or 1 µl/sample of concentrate mouse anti-myc monoclonal [clone 9E10, Developmental Hybridoma Studies Bank (DHSB)]. For western blotting, we used mouse anti-V5 monoclonal at 1:1000, rabbit anti-GFP polyclonal (Invitrogen, #A6455) at 1:1000, 9E10 (DHSB) at 1:1000, or mouse anti-actin (Millipore Sigma, #MAB1501) at 1:5000. We used goat anti-mouse IgG secondary antibody DyLight™ 680 conjugated (Rockland, #610-144-002) and goat anti-rabbit IgG secondary antibody DyLight™ 680 conjugated (Rockland, #610-145-002), both at 1:10,000 dilution.

### Hemocyte isolation and immunohistochemistry

Wandering third-instar larvae were washed in 1× phosphate buffered saline (1× PBS). The larvae were then dissected into pap pen wells drawn on Superfrost Plus microscope slides (Thermo Fisher Scientific, catalog # 1255034). To isolate the hemocytes in ‘bleeds’, the larval cuticle was punctured using fine forceps, and the hemolymph was allowed to extrude from the hemocoel into 30 μl 1× PBS. In subsequent steps, microscope slides were kept in humidified chambers. Hemocytes were allowed to settle onto the slide for 30-45 min. Hemocytes were fixed by the addition of 12.5 μl of 16% paraformaldehyde (PFA) into the 30 μl 1× PBS for a final concentration of 4% PFA and incubated for 10 min. The fixative solution was removed manually, and samples were washed twice for 10 min each in 1× PBS-T (0.01% Triton X-100 in 1× PBS). The following steps were performed with mild agitation. Hemocytes were blocked in 10% normal goat serum (NGS; Vector Laboratories, S-1000) in 1× PBS-T for 1 h at room temperature or overnight at 4°C. Primary antibodies were diluted in blocking solution and incubated overnight at 4°C. Samples were washed twice with 1× PBS-T for 10 min. Secondary antibodies were diluted in blocking solution and incubated for 2 h at room temperature. Hemocytes were washed twice with 1× PBS-T for 10 min. Samples were mounted in VectaShield (Vector Laboratories, H-1000). Images of the samples were captured using a Zeiss LSM510 confocal microscope at 20×, 40× or 63× magnification.

### Hemocyte counts

To count circulating hemocytes, staged, third-instar larvae were washed in 1× PBS and then the hemolymph was bled to 20 μl 1× PBS, and 10 μl of the total volume was transferred onto a hemocytometer. The total number of cells were counted, multiplied by the original volume (20 µl) and the average number of hemocytes per larva was calculated. At least 15 larvae of each genotype were counted, and the counting was carried out in triplicates. We plotted the average number of hemocytes/larva and the percentage of lamellocytes using GraphPad Prism7. We used the Student's *t*-test to determine statistical significance.

### Microtumors

Microtumors were classified as aggregates of hemocytes that contain lamellocytes (as defined by F-actin, L1 and/or morphology), that were not melanized and that were at least 50 µm in size.

### Hemocyte aggregation assay

The hemocyte aggregation assay is essentially a procedure to quantify microtumors from the entire hemolymph of a single larva. Wandering third-instar larvae were washed in 1× PBS. Hemocytes from a single larva were bled into 5 μl 1× PBS in black resin dissection dishes and allowed to settle for 30 min in a humidifier chamber. Images were taken using a Nikon D5100 camera mounted on a Nikon SMZ 1500 dissecting microscope with UV X-cite 120 at 5× magnification. ImageJ was used to measure the GFP intensity in each sample. We determined that the GFP intensity within the cells does not change between genotypes. GFP intensities were normalized to the control, with the control value set at 1. The relative GFP intensity was plotted on the *y*-axis as ‘hemocyte aggregation’. These values were graphed and analyzed using GraphPad Prism 7 and statistical analysis was assessed by two-way ANOVA.

### Hemocyte isolation for qPCR and immunoprecipitation

Larvae were washed in 1× PBS. Hemocytes were collected into 100 µl 1× PBS droplet/well on dissection plates. These cells were then transferred into Eppendorf tubes and kept on ice. To separate hemocytes from the hemolymph, bleeds were centrifuged at 4°C at 1500 rpm (200 ***g***) for 10 min, and the supernatant was discarded. mRNA or protein extraction was performed on hemocyte pellets following the procedure below.

### mRNA extraction and qPCR from hemocytes

mRNA was extracted from hemocyte pellets by homogenizing them into TRIzol (Thermo Fisher Scientific), followed by chloroform extraction and ethanol precipitation. The mRNAs were diluted into H_2_O. A total of 0.5-1 µg mRNA was treated with DNase (Ambion) as per the manufacturer's instructions. The mRNA was reverse transcribed using a Maxima reverse transcriptase kit (Thermo Fisher Scientific). qPCR was performed using a SYBR Green PCR Master Mix kit (Thermo Fisher Scientific), using primers from Integrated DNA Technologies (described below) in a CFX96 Touch™ Real-Time PCR Detection System, and using the Bio-Rad CFX Manager 3.1. We used 95°C for denaturing for 10 s, 65°C for primer annealing for 30 s, and 72°C for primer extension for 10 s. These steps were repeated 40 times. ΔΔCt for each gene was calculated relative to the expression of the respective gene from the *HaHmlLT>GFP* control cross and normalized to the *Rpl15* endogenous control. Normalized relative gene expression was plotted in GraphPad Prism7 and Student's *t*-test was used for statistical analysis per gene for each genotype compared to the control genotype.

### Primers

Primers for the indicated genes (Table S1) were manufactured by Integrated DNA Technologies using the following settings: primers must expand exon junctions, 50% CG, product 100 to 200 bp in length, and melting temperature of 65°C.

### Immunoprecipitation of hemocytes

After centrifugation (see mRNA extraction section above), hemocyte pellets were lyzed using 100 µl of an NP-40 lysis buffer (10 mM HEPES, 10 mM KCl, 1 mM EDTA, 100 µM EGTA, 1 mM NaOV, 10 mM β-glycerophosphate, 100 mM NaF, 1.05× cOmplete protease inhibitor, 1% NP-40). Cells were lysed by pipetting up and down, incubated on ice for 7 min and centrifuged at 4°C for 10 min at 10,000 rpm (6149 ***g***). The supernatant was transferred to a new tube. To isolate sufficient quantities of hemocytes, we dissected larvae each day, flash freezing the hemocyte lysate and storing it at −20°C until enough larvae were collected to perform an immunoprecipitation (typically 20-55 larvae). Hemocyte lysates were then pooled together and lysate buffer was added to bring the total volume to 500 µl. We ran 4% of the total lysate for the input control. A total of 500 µl of lysate buffer was added to the rest of the lysate and used for immunoprecipitation.

For each sample, we added 20 µl of a 50:50 slurry of Protein A-Sepharose beads (GE Healthcare, #17-5280-01) in lysis buffer, and 1 µl of mouse anti-V5 monoclonal per sample, and incubated at 4°C overnight with agitation. Samples were then centrifuged at 4°C for 1 min at 7000 rpm (3013 ***g***), and the flow through was discarded. The samples were washed four times with wash buffer (10 mM HEPES, 10 mM KCl, 1 mM EDTA, 100 µM EGTA, 1% NP-40) and centrifuged at 4°C for 1 min at 7000 rpm. After the last wash, all excess supernatant was removed using a 50 µl Hamilton syringe. Immunoprecipitated proteins were eluted by adding 20 µl of 2× Laemmli [containing β-mercaptoethanol (βME)] in lysis buffer and centrifuging for 1 min at 7000 rpm at room temperature. Samples were boiled for 5 min and centrifuged for 1 min at 7000 rpm at room temperature. The eluate was removed using a Hamilton syringe and transferred to a new tube for SDS-PAGE and western blotting.

### S2 cell culture, transfection and KDACi treatment

S2 cells were grown in Schneider media (10% FBS and 1% Pen/Strep). S2 cells were transfected using the Qiagen Quick-Start Protocol (Qiagen, #301427). Briefly, 2.4×10^6^ S2 cells were seeded in 10 mm plates with 9.6 ml of Schneider media a day prior to transfection. We transfected 12 μg of *Act5c-hop-myc-his* plasmid (Ekas et al., 2010) using the Qiagen protocol (96 µl Enhancer and 60 µl Effectene in 3.6 ml Schneider media). At 32 h post-transfection, KDACi were added to the cultures for 16 h of treatment as described (Klampfer et al., 2004). After a total of 48 h of transfection, the experiment was terminated. S2 cells were collected by centrifuging them in a 15 ml conical tube for 3 min at 1000 ***g***. S2 cells were lysed using 1 ml of 1% NP-40 lysis buffer (10 mM HEPES, 10 mM KCl, 1 mM EDTA, 100 µM EGTA, 1 mM NaOV, 10 mM β-glycerophosphate, 100 mM NaF, 1.05× cOmplete protease inhibitor, 1% NP-40) and incubated on ice for 7 min and centrifuged at 4°C for 10 min at 10,000 rpm. The supernatant was transferred to a new tube. 5% of the total lysate was saved in sample buffer with βME (250 mM Tris, pH 6.8, 25% glycerol, 2% SDS, 5% βME) for input control. Input samples were boiled for 5 min and centrifuged at 15,000 rpm (17,530 ***g***) for 1 min before freezing or loading them onto an SDS-PAGE gel (see below). The rest of the lysate was used for immunoprecipitation as described above for hemocytes using 1 µl of 9E10 (mouse anti-myc monoclonal) per sample. TSA was a gift from Dr David Levy (NYU School of Medicine, USA) and NaBut was a gift from Dr Danny Reinberg (NYU School of Medicine, USA).

### Western blotting

We cast by hand 8%, 12% or 15% standard SDS-PAGE gels. Proteins were separated by electrophoresis using 1× SDS-Tris running buffer (0.3% Tris-base, 1.44% glycine, 0.1% SDS, 8.5 pH). Proteins were transferred to 0.45 µm nitrocellulose membrane (Bio-Rad, #1620115) by western blotting at 300 mAmp for 2 h at 4°C in 1× transfer buffer (0.3% Tris-base, 1.44% glycine, 20% methanol). Each subsequent step was performed with mild agitation. Membranes were blocked with 5% non-fat dairy milk in 1× TBS-T [0.242% Tris-base, 0.8% NaCl (Fisher Scientific, #BP358-1), 0.1% Tween (Fisher Scientific, #BP337-500)] for 1-2 h at room temperature or overnight at 4°C. Excess block was removed by quickly washing the membrane in 1× TBS-T. Membranes were blotted with respective primary antibodies in 1× TBS-T for at least 1 h at room temperature or overnight at 4°C. Membranes were then washed three times for 10 min with 1× TBS-T. Secondary antibodies were diluted in 1× TBS-T and incubated for at least 1 h at room temperature. Membranes were then washed at least three times for 10 min with 1× TBS-T and imaged using a Li-Cor Odyssey scanner with the Odyssey Infrared Imaging System Application Software, Version 3.0. We used ImageJ to calculate the density of the protein bands on western blots. We normalized the intensity of the Hop-GFP-V5 band to that of the GFP band. We normalized the intensity of the Hop-Myc-His band to that of the actin band for the S2 cell experiments. We plotted the relative intensity of the Hop-GFP-V5 bands or the Hop-Myc-His bands using GraphPad Prism7. To determine statistical significance, we used Student's *t*-test for the *in vivo* Hop-GFP-V5 experiment and one-way ANOVA for the *in vitro* Hop-Myc-His experiment.

### Male survival

We quantified the number of adult *hop^Tum^*/Y males and adult *hop^Tum^*/+ females for all genotypes. Survival was calculated as the number of eclosed *hop^Tum^* males divided by the total number of eclosed animals bearing the *hop^Tum^* chromosome.

## Supplementary Material

Supplementary information
