# Supplementary material for: Enhancer of Polycomb and the Tip60 complex repress hematological tumor initiation by negatively regulating JAK/STAT pathway activity
Source: Dis Model Mech. 2019 May 30;12(5):dmm038679. doi: 10.1242/dmm.038679 (PMC6550037; doi:10.1242/dmm.038679)
Supplement: Supplementary information [file dmm-12-038679-s1.pdf]

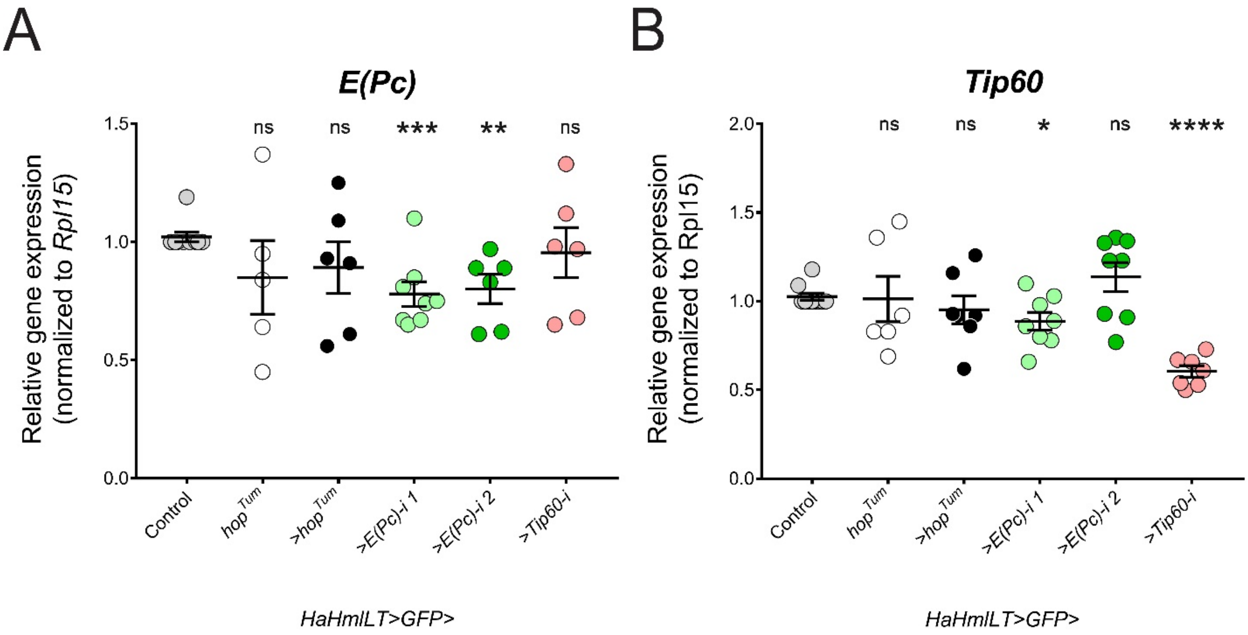

Figure S1: Significant knockdown of *E(Pc)* or *Tip60* in hemocytes

(A) Compared to the control (gray circles), *E(Pc)* is significantly decreased in hemocytes from *HaHmlLT>GFP>E(Pc) RNAi* (using either RNAi construct, light green or dark green circles), and but not in hemocytes from endogenous *hop*^{Tum} (white circles), *HaHmlLT>GFP>hop*^{Tum} (black circles), or *HaHmlLT>GFP>Tip60 RNAi* (pink circles) compared to the control.

(B) Compared to the control (gray circles), *Tip60* (B) is significantly in hemocytes from *HaHmlLT>GFP>Tip60 RNAi* (pink circles) but not in endogenous *hop*^{Tum} (white circles), *HaHmlLT>GFP>hop*^{Tum} (black circles), *HaHmlLT>GFP>E(Pc) RNAi* (using either RNAi construct, light green and dark green circles) compared to the control.

* indicates P<0.05; ** indicates P<0.01; *** indicates P<0.001; **** indicates P<0.0001. "ns" is not significant.

Disease Models & Mechanisms • Supplementary information

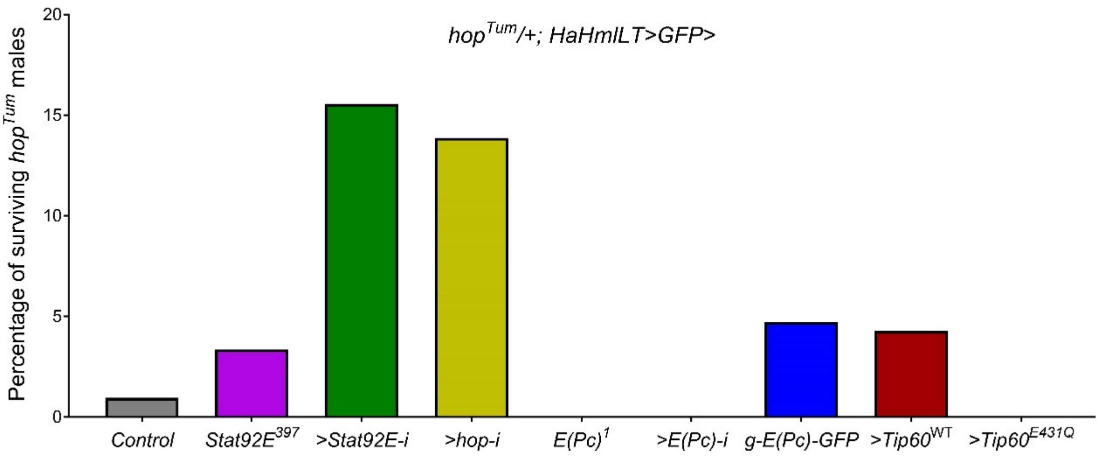

	Control	*Stat92E*^{397}	>*Stat92E-i*	>*hop-i*	*E(Pc)*^{1}	>*E(Pc)-i*	*g-E(Pc)-GFP*	>*Tip60*^{WT}	>*Tip60*^{E431Q}
Total number of animals	528	388	90	310	434	203	148	117	58
Total number of hop^{Tum} males	5	13	14	43	0	0	7	5	0
Percentage of hop^{Tum} males	0.95%	3.35%	15.56%	13.87%	0.00%	0.00%	4.73%	4.27%	0.00%

Figure S2. Increasing expression of wild-type *E(Pc)* or wild-type *Tip60* rescues the lethality of *hop*^{Tum}/Y males

Graph indicates the percentage of *hop*^{Tum}*/Y* males out of the total number of offsrping that carry the *hop*^{Tum} chromosome (i.e, *hop*^{Tum}*/Y* males and *hop*^{Tum}/+ females). *hop*^{Tum}*/Y* males are rarely observed in the control outcross (gray bar). *hop*^{Tum}*/Y* males are observed with higher frequency when the animal is heterozygous for *Stat92E* (purple bar) or when *Stat92E* or *hop* are hematopoietically depleted (green and yellow bars, respectively). *hop*^{Tum}*/Y* males are also observed at a higher rate when animals have increased systemic levels of *E(Pc)* (blue bar) or increased hematopoietic expression of wild-type *Tip60* (red bar). By contrast, *hop*^{Tum}*/Y* males are never observed when the systemic or hematopoietic dose of E(Pc) (labelled "*E(Pc)*^{1}" or "*E(Pc)-i*") or of Tip60 (labelled *Tip60*^{E341Q}) is reduced.

Genotypes in all figures

Figure 1:

(D)

hop^{Tum}/+; Hand-gal4, HmlΔ-Gal4, UAS-FLP.JD1, UAS-2xEGFP; Gal4-Act5C (FRT.CD2) (Control)

hop^{Tum}/w; Hand-gal4, HmlΔ-Gal4, UAS-FLP.JD1, UAS-2xEGFP/+; Gal4-Act5C (FRT.CD2)/Stat92E^{397}

hop^{Tum}/w; Hand-gal4, HmlΔ-Gal4, UAS-FLP.JD1, UAS-2xEGFP/(CyO or Sp); Gal4-Act5C (FRT.CD2)/UAS-dome$^{\Delta CYT}$

(E)

hop^{Tum}/+; +/+; +/+ (Control)

hop^{Tum}/w; +/+; Stat92E^{397}/+

hop^{Tum}/upd2$^{\Delta}$, upd3$^{\Delta}$; +/+; +/+

Figure 2:

hop^{Tum}/+; +/+; +/+ (Control)

hop^{Tum}/w; +/+; Stat92E^{397}/+

hop^{Tum}/w; ED2219/+; +/+

hop^{Tum}/w; BSC703/ +;+/+

hop^{Tum}/w; BSC336/ +;+/+

hop^{Tum}/w; BSC304/ +;+/+

hop^{Tum}/w; BSC358/ +;+/+

hop^{Tum}/w; BSC231/ +;+/+

hop^{Tum}/w; ED2222/ +;+/+

hop^{Tum}/+; cn, E(Pc)^{1}, bw^{1} / +;+/+

hop^{Tum}/y, w; E(Pc)^{w3}/ +;+/+

hop^{Tum}/+; inv^{30}/ +;+/+

hop^{Tum}/y, w; inv^{KG04405}/ +;+/+

hop^{Tum}/f^{36a}; inv^{E}en^{E}/ +;+/+

hop^{Tum}/+; en^{1}/ +;+/+

hop^{Tum}/+; en^{4}/ +;+/+

hop^{Tum}/+; en^{54}/ +;+/+

hop^{Tum}/+; en^{59}/ +;+/+

hop^{Tum}/w; tou^{1}/ +;+/+

hop^{Tum}/w; tou^{2}/ +;+/+

hop^{Tum}/w+; tou^{KG02432}/ +;+/+

Figure 3:

(A)

hop^{Tum}/+; Hand-gal4, HmlΔ-Gal4, UAS-FLP.JD1, UAS-2xEGFP/+; Gal4-Act5C (FRT.CD2)/+ (Control)
hop^{Tum}/w; Hand-gal4, HmlΔ-Gal4, UAS-FLP.JD1, UAS-2xEGFP/+; Gal4-Act5C (FRT.CD2)/Stat92E^{397}
hop^{Tum}/w; Hand-gal4, HmlΔ-Gal4, UAS-FLP.JD1, UAS-2xEGFP/ UAS-E(Pc)-RNAi 1 (GD12282); Gal4-Act5C (FRT.CD2)/+
hop^{Tum}/y, v; Hand-gal4, HmlΔ-Gal4, UAS-FLP.JD1, UAS-2xEGFP/+; Gal4-Act5C (FRT.CD2)/UAS-E(Pc)-RNAi 2 (JF03101)
hop^{Tum}/y, v; Hand-gal4, HmlΔ-Gal4, UAS-FLP.JD1, UAS-2xEGFP/+; Gal4-Act5C (FRT.CD2)/UAS-Tip60-RNAi (HM05049)
(B)
w, UAS-hop^{Tum2M}/+; Hand-gal4, HmlΔ-Gal4, UAS-FLP.JD1, UAS-2xEGFP/+; Gal4-Act5C (FRT.CD2)/+ (Control)
w, UAS-hop^{Tum2M}/w; Hand-gal4, HmlΔ-Gal4, UAS-FLP.JD1, UAS-2xEGFP/ UAS-E(Pc)-RNAi 1 (GD12282); Gal4-Act5C (FRT.CD2)/+
w, UAS-hop^{Tum2M}/y, v; Hand-gal4, HmlΔ-Gal4, UAS-FLP.JD1, UAS-2xEGFP/+; Gal4-Act5C (FRT.CD2)/UAS-Tip60-RNAi (HM05049)
(C)
hop^{Tum}/+; Hand-gal4, HmlΔ-Gal4, UAS-FLP.JD1, UAS-2xEGFP/+; Gal4-Act5C (FRT.CD2)/+ (Control)
hop^{Tum}/w; Hand-gal4, HmlΔ-Gal4, UAS-FLP.JD1, UAS-2xEGFP/+; Gal4-Act5C (FRT.CD2)/Stat92E^{397}
hop^{Tum}/w; Hand-gal4, HmlΔ-Gal4, UAS-FLP.JD1, UAS-2xEGFP/E(Pc)^{1}
hop^{Tum}/w; Hand-gal4, HmlΔ-Gal4, UAS-FLP.JD1, UAS-2xEGFP/+; Gal4-Act5C (FRT.CD2)/genomic-E(Pc)-GFP
hop^{Tum}/+; Hand-gal4, HmlΔ-Gal4, UAS-FLP.JD1, UAS-2xEGFP/+; Gal4-Act5C (FRT.CD2)/UAS-E(Pc)-GFP
(D)
hop^{Tum}/+; Hand-gal4, HmlΔ-Gal4, UAS-FLP.JD1, UAS-2xEGFP/+; Gal4-Act5C (FRT.CD2)/+ (Control)
hop^{Tum}/w; Hand-gal4, HmlΔ-Gal4, UAS-FLP.JD1, UAS-2xEGFP/+; Gal4-Act5C (FRT.CD2)/Stat92E^{397}
hop^{Tum}/w; Hand-gal4, HmlΔ-Gal4, UAS-FLP.JD1, UAS-2xEGFP/+; Gal4-Act5C (FRT.CD2)/UAS-Tip60^{WT}
hop^{Tum}/w; Hand-gal4, HmlΔ-Gal4, UAS-FLP.JD1, UAS-2xEGFP/+; Gal4-Act5C (FRT.CD2)/UAS-Tip60^{E431Q}

Figure 4
(A-B) *w*/+; *Hand-gal4, HmlΔ-Gal4, UAS-FLP.JD1, UAS-2xEGFP/+; Gal4-Act5C (FRT.CD2)/+* (Control)

(C-D) *UAS-hop^{Tum2M}/w; Hand-gal4, HmlΔ-Gal4, UAS-FLP.JD1, UAS-2xEGFP/(+; +)*
(E-F) *w/y, v*; *Hand-gal4, HmlΔ-Gal4, UAS-FLP.JD1, UAS-2xEGFP/+; Gal4-Act5C (FRT.CD2)/UAS-E(Pc)-RNAi 2 (JF03101)*
(G-H) *w/y, v; Hand-gal4, HmlΔ-Gal4, UAS-FLP.JD1, UAS-2xEGFP/+; Gal4-Act5C (FRT.CD2)/UAS-Tip60-RNAi (HM05049)*
(I-J) *w/w; Hand-gal4, HmlΔ-Gal4, UAS-FLP.JD1, UAS-2xEGFP/+; Gal4-Act5C (FRT.CD2)/UAS-Tip60^{E431Q}*
(K)
w/+; Hand-gal4, HmlΔ-Gal4, UAS-FLP.JD1, UAS-2xEGFP/+; Gal4-Act5C (FRT.CD2)/+ (Control)
w/w; Hand-gal4, HmlΔ-Gal4, UAS-FLP.JD1, UAS-2xEGFP/ UAS-E(Pc)-RNAi 1 (GD12282); Gal4-Act5C (FRT.CD2)/+
w/y, w; Hand-gal4, HmlΔ-Gal4, UAS-FLP.JD1, UAS-2xEGFP/+; Gal4-Act5C (FRT.CD2)/UAS-Tip60-RNAi (HM05049)
w/+; Hand-gal4, HmlΔ-Gal4, UAS-FLP.JD1, UAS-2xEGFP/+; Gal4-Act5C (FRT.CD2)/UAS-Tip60^{E431Q}
(L)
w/+; Hand-gal4, HmlΔ-Gal4, UAS-FLP.JD1, UAS-2xEGFP/+; Gal4-Act5C (FRT.CD2)/+ (Control)
w, UAS-hop^{Tum2M}/+; Hand-gal4, HmlΔ-Gal4, UAS-FLP.JD1, UAS-2xEGFP/+ ; Gal4-Act5C (FRT.CD2)/+
w/y, v; Hand-gal4, HmlΔ-Gal4, UAS-FLP.JD1, UAS-2xEGFP/+; Gal4-Act5C (FRT.CD2)/UAS-E(Pc)-RNAi 2 (JF03101)
(M)
w/+; Hand-gal4, HmlΔ-Gal4, UAS-FLP.JD1, UAS-2xEGFP/+; Gal4-Act5C (FRT.CD2)/+ (Control)
w, UAS-hop^{Tum2M}/+; Hand-gal4, HmlΔ-Gal4, UAS-FLP.JD1, UAS-2xEGFP/+
w/y, v; Hand-gal4, HmlΔ-Gal4, UAS-FLP.JD1, UAS-2xEGFP/+; Gal4-Act5C (FRT.CD2)/UAS-E(Pc)-RNAi 2 (JF03101)
(N) *w/+; Hand-gal4, HmlΔ-Gal4, UAS-FLP.JD1, UAS-2xEGFP/+*
(O) *w/w*; *Hand-gal4, HmlΔ-Gal4, UAS-FLP.JD1, UAS-2xEGFP+; Gal4-Act5C (FRT.CD2)/UAS-Bap55-RNAi (GD11955)*
(P) *w/y, sc, v*; *Hand-gal4, HmlΔ-Gal4, UAS-FLP.JD1, UAS-2xEGFP/+; Gal4-Act5C (FRT.CD2)/ UAS-dom-RNAi (HMS02208)*

<u>Figure 5</u>
(A)
w/+; Hand-gal4, HmlΔ-Gal4, UAS-FLP.JD1, UAS-2xEGFP/+; Gal4-Act5C (FRT.CD2)/+ (Control)

Disease Models & Mechanisms • Supplementary information

w, UAS-hop^{Tum2M}/+; Hand-gal4, HmlΔ-Gal4, UAS-FLP.JD1, UAS-2xEGFP/+; Gal4-Act5C (FRT.CD2)/+
w, UAS-hop^{Tum2M}/+; Hand-gal4, HmlΔ-Gal4, UAS-FLP.JD1, UAS-2xEGFP/UAS-Stat92E-RNAi (GD4922); Gal4-Act5C (FRT.CD2)/+
w, UAS-hop^{Tum2M}/+; Hand-gal4, HmlΔ-Gal4, UAS-FLP.JD1, UAS-2xEGFP/+; Gal4-Act5C (FRT.CD2)/UAS-hop-RNAi (GL00305)
(B)
hop^{Tum}/+; Hand-gal4, HmlΔ-Gal4, UAS-FLP.JD1, UAS-2xEGFP/+; Gal4-Act5C (FRT.CD2)/+ (Control)
hop^{Tum}/w; Hand-gal4, HmlΔ-Gal4, UAS-FLP.JD1, UAS-2xEGFP/+; Gal4-Act5C (FRT.CD2)/Stat92E^{397}
hop^{Tum}/w; Hand-gal4, HmlΔ-Gal4, UAS-FLP.JD1, UAS-2xEGFP/ UAS-Stat92E-RNAi (GD4922); Gal4-Act5C (FRT.CD2)/+
hop^{Tum}/y, sc, v; Hand-gal4, HmlΔ-Gal4, UAS-FLP.JD1, UAS-2xEGFP/+; Gal4-Act5C (FRT.CD2)/UAS-hop RNAi (GL00305)
(C)
w/+; Hand-gal4, HmlΔ-Gal4, UAS-FLP.JD1, UAS-2xEGFP/+; Gal4-Act5C (FRT.CD2)/+ (Control)
w/w; Hand-gal4, HmlΔ-Gal4, UAS-FLP.JD1, UAS-2xEGFP/ UAS-Stat92E-RNAi (GD4922); Gal4-Act5C (FRT.CD2)/+
w/y, v; Hand-gal4, HmlΔ-Gal4, UAS-FLP.JD1, UAS-2xEGFP/+; Gal4-Act5C (FRT.CD2)/UAS-E(Pc)-RNAi 2 (JF03101)
w/w; Hand-gal4, HmlΔ-Gal4, UAS-FLP.JD1, UAS-2xEGFP/+; UAS-Stat92E-RNAi (GD4922); Gal4-Act5C (FRT.CD2)/UAS-E(Pc)-RNAi 2 (JF03101)
(D)
w/+; Hand-gal4, HmlΔ-Gal4, UAS-FLP.JD1, UAS-2xEGFP/+; Gal4-Act5C (FRT.CD2)/+ (Control)
w/w; Hand-gal4, HmlΔ-Gal4, UAS-FLP.JD1, UAS-2xEGFP/ UAS-Stat92E-RNAi (GD4922); Gal4-Act5C (FRT.CD2)/+
w/y, v; Hand-gal4, HmlΔ-Gal4, UAS-FLP.JD1, UAS-2xEGFP/+; Gal4-Act5C (FRT.CD2)/UAS-E(Pc)-RNAi 2 (JF03101)
w/w; Hand-gal4, HmlΔ-Gal4, UAS-FLP.JD1, UAS-2xEGFP/+; UAS-Stat92E-RNAi (GD4922); Gal4-Act5C (FRT.CD2)/UAS-E(Pc)-RNAi 2 (JF03101)
(E)
w/+; Hand-gal4, HmlΔ-Gal4, UAS-FLP.JD1, UAS-2xEGFP/+; Gal4-Act5C (FRT.CD2)/+ (Control)
w/y, sc, v; Hand-gal4, HmlΔ-Gal4, UAS-FLP.JD1, UAS-2xEGFP/+; Gal4-Act5C (FRT.CD2)/UAS-hop-RNAi (GL00305)
w/w; Hand-gal4, HmlΔ-Gal4, UAS-FLP.JD1, UAS-2xEGFP/ UAS-E(Pc)-RNAi 1 (GD12282); Gal4-Act5C (FRT.CD2)/+

w/w; Hand-gal4, HmlΔ-Gal4, UAS-FLP.JD1, UAS-2xEGFP/ UAS-E(Pc)-RNAi 1 (GD12282); Gal4-Act5C (FRT.CD2)/UAS-hop-RNAi (GL00305)
(F)
w/+; Hand-gal4, HmlΔ-Gal4, UAS-FLP.JD1, UAS-2xEGFP/+; Gal4-Act5C (FRT.CD2)/+
(Control)
w/y, sc, v; Hand-gal4, HmlΔ-Gal4, UAS-FLP.JD1, UAS-2xEGFP/+; Gal4-Act5C (FRT.CD2)/UAS-hop-RNAi (GL00305)
w/w; Hand-gal4, HmlΔ-Gal4, UAS-FLP.JD1, UAS-2xEGFP/ UAS-E(Pc)-RNAi 1 (GD12282); Gal4-Act5C (FRT.CD2)/+
w/w; Hand-gal4, HmlΔ-Gal4, UAS-FLP.JD1, UAS-2xEGFP/ UAS-E(Pc)-RNAi 1 (GD12282); Gal4-Act5C (FRT.CD2)/UAS-hop-RNAi (GL00305))

Figure 6
(A) *w/+; 10X Stat92E-DsRed/+; +/+*
(D) *hop^{Tum}/w; 10XStat92E-DsRed/+ ; +/+*
(C) *w/ y, w, $hs\text{-}flp^{122}$; Hand-gal4, HmlΔ-Gal4, UAS-FLP.JD1, UAS-2xEGFP/10X Stat92E-DsRed; Gal4-Act5C (FRT.CD2)/UAS-E(Pc)-RNAi 2 (JF03101)*
(D-H)
w/+; Hand-gal4, HmlΔ-Gal4, UAS-FLP.JD1, UAS-2xEGFP/+; Gal4-Act5C (FRT.CD2)/+
(Control)
hop^{Tum}/+; Hand-gal4, HmlΔ-Gal4, UAS-FLP.JD1, UAS-2xEGFP/+; Gal4-Act5C (FRT.CD2)/+
w,UAS-hop^{Tum2M}/+; Hand-gal4, HmlΔ-Gal4, UAS-FLP.JD1, UAS-2xEGFP/+; Gal4-Act5C (FRT.CD2)/+
w/w; Hand-gal4, HmlΔ-Gal4, UAS-FLP.JD1, UAS-2xEGFP/UAS-E(Pc)-RNAi 1 (GD12282); Gal4-Act5C (FRT.CD2)/+
w/y,v; Hand-gal4, HmlΔ-Gal4, UAS-FLP.JD1, UAS-2xEGFP/+; Gal4-Act5C (FRT.CD2)/UAS-E(Pc)-RNAi 2 (JF03101)
w/y,v; Hand-gal4, HmlΔ-Gal4, UAS-FLP.JD1, UAS-2xEGFP/+; Gal4-Act5C (FRT.CD2)/UAS-Tip60-RNAi (HM05049)

Figure 7
(A)
w/+; Hand-gal4, HmlΔ-Gal4, UAS-FLP.JD1, UAS-2xEGFP/+; Gal4-Act5C (FRT.CD2)/+
(Control)
w/y, w; Hand-gal4, HmlΔ-Gal4, UAS-FLP.JD1, UAS-2xEGFP/UAS-E(Pc)-RNAi 1 (GD12282); Gal4-Act5C (FRT.CD2)/hop-GFP-V5
(B)
w/w; Hand-gal4, HmlΔ-Gal4, UAS-FLP.JD1, UAS-2xEGFP/+; Gal4-Act5C (FRT.CD2)/+
(Control)

Disease Models & Mechanisms • Supplementary information

w/y; Hand-gal4, HmlΔ-Gal4, UAS-FLP.JD1, UAS-2xEGFP/UAS-E(Pc)-RNAi 1 (GD12282); Gal4-Act5C (FRT.CD2)/hop-GFP-V5

(C)

w/+; Hand-gal4, HmlΔ-Gal4, UAS-FLP.JD1, UAS-2xEGFP/+; Gal4-Act5C (FRT.CD2)/+
(Control)

hop^{Tum}/+; Hand-gal4, HmlΔ-Gal4, UAS-FLP.JD1, UAS-2xEGFP/+; Gal4-Act5C (FRT.CD2)/+

w, UAS-hop^{Tum2M}/+; Hand-gal4, HmlΔ-Gal4, UAS-FLP.JD1, UAS-2xEGFP/+; Gal4-Act5C (FRT.CD2)/+

w/w; Hand-gal4, HmlΔ-Gal4, UAS-FLP.JD1, UAS-2xEGFP/UAS-E(Pc)-RNAi 1 (GD12282); Gal4-Act5C (FRT.CD2)/+

w/y, v; Hand-gal4, HmlΔ-Gal4, UAS-FLP.JD1, UAS-2xEGFP/+; Gal4-Act5C (FRT.CD2)/UAS-E(Pc)-RNAi 2 (JF03101)

w/y, v; Hand-gal4, HmlΔ-Gal4, UAS-FLP.JD1, UAS-2xEGFP/+; Gal4-Act5C (FRT.CD2)/UAS-Tip60-RNAi (HM05049)

<u>Figure S1</u>

(A-B)

w/+; Hand-gal4, HmlΔ-Gal4, UAS-FLP.JD1, UAS-2xEGFP/+; Gal4-Act5C (FRT.CD2)/+
(Control)

hop^{Tum}/+; Hand-gal4, HmlΔ-Gal4, UAS-FLP.JD1, UAS-2xEGFP/+; Gal4-Act5C (FRT.CD2)/+

w,UAS-hop^{Tum2M}/+; Hand-gal4, HmlΔ-Gal4, UAS-FLP.JD1, UAS-2xEGFP/+; Gal4-Act5C (FRT.CD2)/+

w/w; Hand-gal4, HmlΔ-Gal4, UAS-FLP.JD1, UAS-2xEGFP/UAS-E(Pc)-RNAi 1 (GD12282); Gal4-Act5C (FRT.CD2)/+

w/y, v; Hand-gal4, HmlΔ-Gal4, UAS-FLP.JD1, UAS-2xEGFP/+; Gal4-Act5C (FRT.CD2)/UAS-E(Pc)-RNAi 2 (JF03101)

w/y, v; Hand-gal4, HmlΔ-Gal4, UAS-FLP.JD1, UAS-2xEGFP/+; Gal4-Act5C (FRT.CD2)/UAS-Tip60-RNAi (HM05049)

<u>Figure S2</u>

hop^{Tum}/+; Hand-gal4, HmlΔ-Gal4, UAS-FLP.JD1, UAS-2xEGFP/+; Gal4-Act5C (FRT.CD2)/+ (Control)

hop^{Tum}/w; Hand-gal4, HmlΔ-Gal4, UAS-FLP.JD1, UAS-2xEGFP/+; Gal4-Act5C (FRT.CD2)/ $Stat92E^{397}$

hop^{Tum}/w; Hand-gal4, HmlΔ-Gal4, UAS-FLP.JD1, UAS-2xEGFP/UAS-Stat92E-RNAi (GD4922);Gal4-Act5C (FRT.CD2)/+

hop^{Tum}/y, sc, v; Hand-gal4, HmlΔ-Gal4, UAS-FLP.JD1, UAS-2xEGFP/+; Gal4-Act5C (FRT.CD2)/UAS-hop-RNAi (GL00305)

hop^{Tum}/+; Hand-gal4, HmlΔ-Gal4, UAS-FLP.JD1, UAS-2xEGFP/$E(Pc)^1$; Gal4-Act5C (FRT.CD2)/+

Disease Models & Mechanisms • Supplementary information

hop^{Tum}/w; Hand-gal4, HmlΔ-Gal4, UAS-FLP.JD1, UAS-2xEGFP/UAS-E(Pc)-RNAi 1 (GD12282); Gal4-Act5C (FRT.CD2)/+

hop^{Tum}/w; Hand-gal4, HmlΔ-Gal4, UAS-FLP.JD1, UAS-2xEGFP/+;Gal4-Act5C (FRT.CD2)/genomic-E(Pc)-GFP

hop^{Tum}/+; Hand-gal4, HmlΔ-Gal4, UAS-FLP.JD1, UAS-2xEGFP/+; Gal4-Act5C (FRT.CD2)/UAS-Tip60^{WT}

hop^{Tum}/+; Hand-gal4, HmlΔ-Gal4, UAS-FLP.JD1, UAS-2xEGFP/+; Gal4-Act5C (FRT.CD2)/UAS-Tip60^{E431Q}

Disease Models & Mechanisms • Supplementary information

Table S1. Primers

Target name	Forward/Reverse	5' to 3' sequence	Reference
Rpl15	Forward	AGGATGCACTTATGGCAAGC	(Grmai et al., 2018)
	Reverse	GCGCAATCCAATACGAGTTC	
Tip60	Forward	CTGGGTTTGCTGTCCTATCG	
	Reverse	GCATTCGCAGATGTCGTTTATAG	
E(Pc)	Forward	CAGCTCCAACAATCCGTACTT	
	Reverse	TCGCAGCTTGAGCATCTTT	
Socs36E	Forward	GCTGCCAGTCAGCAATATGT	(Flaherty et al., 2009)
	Reverse	GACTGCGGCAGCAACTGT	
chinmo	Forward	ACTGGCCGATGTCATATTATCC	
	Reverse	TGTGGGCGTGTTCTCAAA	
zfh1	Forward	ATACGAGCATTCCGGTCAAC	
	Reverse	GGCACTTGGAGCACTGAA	
upd2	Forward	ACCCTGGAGTACGGCAATCT	(Bazzi et al., 2018)
	Reverse	CTGATCCTTGCGGAACTTGT	
upd3	Forward	CCACAGTGAGCACCAAGACT	(Bazzi et al., 2018)
	Reverse	CAGGTCCCAGTGCAACTTGA	
Stat92E	Forward	CGAGTGCATCATAATGTCAGAGA	
	Reverse	TTTGAATCTCGCCCGATG	
hop #1	Forward	CACACTCAAGTGGATCCTTGT	
	Reverse	TGTAGATACCCAGGTAGGAGATG	
hop #3	Forward	GCATACCCGCCTCCAAATA	
	Reverse	ATCGAATCTGGATACCACTGC	
hop #5	Forward	CTGCAGTGGTATCCAGATTCG	
	Reverse	TCTGTGCCAAGCGAAAGAA	
hop #7	Forward	TCACCACCAACACCAATTCC	
	Reverse	GCCAACAAGGATCCACTTGA	

Disease Models & Mechanisms • Supplementary information
